# Effectiveness of a dietician-led intervention in reducing glycated haemoglobin among people with type 2 diabetes in Nepal: a single centre, open-label, randomised controlled trial

**DOI:** 10.1016/j.lansea.2023.100285

**Published:** 2023-09-25

**Authors:** Dev Ram Sunuwar, Suvash Nayaju, Raja Ram Dhungana, Kshitij Karki, Pranil Man Singh Pradhan, Pramod Poudel, Chitrakala Nepal, Madhu Thapa, Nani Shobha Shakya, Matina Sayami, Pradip Krishna Shrestha, Renu Yadav, Devendra Raj Singh

**Affiliations:** a Department of Public Health, Asian College for Advance Studies, Purbanchal University, Nepal; b Department of Nutritional Sciences, School of Public Health, University of Michigan, Ann Arbor, USA; c Faculty of Medicine Nursing and Health Sciences, Monash University, Australia; d Department of Food, Agriculture, and Bioresources, School of Environment Resources and Development, Asian Institute of Technology, Thailand; e Department of Community Medicine, Maharajgunj Medical Campus, Institute of Medicine, Tribhuvan University, Kathmandu, Nepal; f Department of Global Health and Population, Harvard T H Chan School of Public Health, Boston, MA 02115, USA; g Central Department of Biotechnology, Tribhuvan University, Kathmandu, Nepal; h Department of Nutrition and Dietetics, College of Applied Food and Dairy Technology, Lalitpur, Nepal; i Department of Internal Medicine, Maharajgunj Medical Campus, Institute of Medicine, Tribhuvan University, Kathmandu, Nepal; j Department of Internal Medicine and Endocrinology, Nepal Mediciti Hospital, Lalitpur, Nepal; k Department of Nutrition and Dietetics, Tribhuvan University Teaching Hospital, Kathmandu, Nepal; l Department of Public Health, Central Institute of Science and Technology (CIST) College, Pokhara University, Kathmandu, Nepal; m School of Human and Health Sciences, University of Huddersfield, Huddersfield, United Kingdom

**Keywords:** Dietary approach, Diet counselling, Dietician led, Dietician delivered, Nutrition education, Type 2 diabetes, Nepal

## Abstract

**Background:**

Nutrition education and counselling are considered a cornerstone for the management of type 2 diabetes (T2D). However, there is limited research related to the management of T2D through dietary approach, particularly in low-income and middle-income countries (LMICs) like Nepal. This study assessed the effectiveness of a dietician-led dietary intervention in reducing glycated haemoglobin (HbA1c) levels among people with T2D.

**Methods:**

An open-label, two-armed, hospital-based, randomised controlled trial was conducted at Tribhuvan University Teaching Hospital, Kathmandu, Nepal. Participants were randomly assigned to either dietician-led dietary intervention group (n = 78) or usual care control group (n = 78). People with type 2 diabetes with HbA1c >6.5% and aged 24−64 years were included in the study. The primary outcome was a change in HbA1c level over six months, and secondary outcomes included changes in biochemical and clinical parameters, Problem Areas in Diabetes (PAID) score, diabetic knowledge, dietary adherence, and macronutrient intake level. Data were analysed using an intention-to-treat approach. This trial is registered with ClinicalTrials.gov, NCT04267367.

**Findings:**

Between August 15, 2021 and February 25, 2022, 156 people with type 2 diabetes were recruited for the study, of which 136 participants completed the trial. At six months of follow-up, compared to baseline values, the mean HbA1c (%) level decreased in the intervention group by 0.48 (95% CI: −0.80 to −0.16), while it increased in the control group by 0.22 (95% CI: −0.21 to 0.66). In an adjusted model, the reduction in HbA1c (%) levels for the intervention was 0.61 (95% CI: −1.04 to −0.17; p = 0.006). In addition, fasting blood glucose was decreased by 18.96 mg/dL (95% CI: −36.12 to −1.81; p = 0.031) after the intervention. The intervention resulted in the reduction of BMI, waist and hip circumference, PAID score, dietary adherence, and macronutrient intake in the intervention group compared to the control group.

**Interpretation:**

The dietician-led intervention improved glycaemic control, improved macronutrient intake, and clinical outcomes among people with type 2 diabetes. The dietician-led intervention may be considered for diabetes management in LMICs.

**Funding:**

The research was funded by the University Grants Commission (UGC), Nepal.

**Research in context**

**Evidence before this study**

Previous studies from high-income countries have shown that dietician-delivered nutrition education and counselling was considered an effective strategy for the management of type 2 diabetes. We searched PubMed up to September 21, 2022, using the search terms "type 2 diabetes" OR "raised blood glucose" AND dietary management OR "nutrition education" OR "nutrition counselling" AND "dietician led" OR "dietician delivered" for studies comparing the dietary intervention versus usual care group. We found 23 trials investigating the effect of a dietician-delivered dietary approach on the management of type 2 diabetes. None of these studies were conducted in low-income and middle-income countries (LMICs) specifically Nepal.

**Added value of this study**

To our knowledge, this is the first randomised controlled trial to investigate the effectiveness of a dietician-led dietary approach for the management of type 2 diabetic patients in Nepal. The study explored clinical, biochemical and macronutrient parameters among type 2 diabetic individuals in Nepal. We found that a simple and cost-effective dietary approach provided by a dietician is an effective strategy for glycaemic control, adequate macronutrient intake, and other clinical outcomes among diabetic individuals. These results imply that the nutrition intervention model offered by dieticians is effective in lowering blood glucose levels, improving clinical parameters, and optimizing dietary intake among people with type 2 diabetes. Moreover, this study also highlighted the significance and roles of dieticians in the effective management of diabetes through dietary approaches in both private and public hospitals in LMICs like Nepal.

**Implications of all the available evidence**

The findings of this study are expected to guide policymakers and program designers to develop future policies and interventions for the effective prevention and management of type 2 diabetes. The national Non-communicable Diseases (NCDs) control and prevention program such as Package for Prevention of Essential Non-Communicable Diseases (PEN) should integrate context-specific diabetes education, dietary guidelines, and portion sizes for Nepali food products for the management of diabetes in the context of Nepal. Further studies are necessary to assess the contextual factors that can facilitate the implementation of an evidence-based policy for the management of diabetes in Nepal.

## Introduction

Three in every four individuals with diabetes live in low-income and middle-income countries (LMICs).^1^ Diabetes has been a growing health concern in Nepal, which has an impact on the population's health in terms of both premature mortality and the burden of living with the condition.^2^ According to estimates from the Global Burden of Diseases, Injuries, and Risk Factors Study (GBD) 2021, the age standardised disability-adjusted life-years (DALY) rate for diabetes in Nepal was 1240.2 (1009.1−1516.9) per 100,000, the years of life lost (YLL) rate was 660.6 (514.9–814.5) per 100,000, and the years lived with disability (YLD) rate was 579.7 (405.0–806.6) per 100,000 in Nepal.^3^ Recent evidence suggests that the prevalence of diabetes in Nepal has doubled from 3% to 6% between 2013 and 2018.^4^^,^^5^

Rapid urbanisation and shifts toward a sedentary lifestyle have had an impact on the rapid rise in diabetes prevalence in many nations, and more prominently in LMICs.^6^^,^^7^ The evidence shows that a low fibre diet with a high glycaemic index, saturated fat intake, specific fatty acids, alcohol consumption and soft drinks are considered poor dietary behaviours associated with diabetes.^8^

It is well known that understanding nutrition-related information is essential for facilitating lifestyle changes.^9^ For example, eating a balanced diet is crucial for recovery from type 2 diabetes (T2D).^10^^,^^11^ Dietary management through nutrition education and counselling could be an important part of diabetes management.^10^, ^11^, ^12^ Evidence suggests that individualised nutrition education and counselling could improve adherence to diet recommendations and glycaemic control.^13^ In addition, dietician-led nutrition education and counselling could have a larger impact on reducing body weight and glucose-related outcomes.^14^, ^15^, ^16^ However, there is limited evidence related to the impact of dietary approach strategies, particularly dietary interventions led by a dietician in LMICs, including Nepal. Despite the policy for the requirements of dieticians in the hospitals,^17^ a very limited number of the hospital have appointed dieticians so far in Nepal. In addition, the role of the dietician in the clinical setting for the management of diabetes has been overlooked by concerned authorities. Also, clinicians prefer to treat diabetes with pharmacological approaches and dietary approaches for managing diabetes has received less attention in the Nepalese context. The dietician delivered context-specific diabetic education, food based dietary guidelines, and portion size for Nepali food items are lacking for the management of diabetes. There is absence of clear guidelines and protocols for dietary strategies for the management of T2D in Nepal. The current study aimed to investigate the effect of a dietician-led dietary approach in the management of diabetes in a tertiary care hospital in Nepal.

## Methods

### Study design and participants

This was a two-armed randomised control trial designed to evaluate the effectiveness of the dietician-led dietary intervention. The study was conducted in the outpatient department of Tribhuvan University Teaching Hospital, Institute of Medicine, Tribhuvan University, Kathmandu, Nepal. It is a tertiary referral centre in Nepal that offers comprehensive medical services. Under the supervision of doctors or clinical officers, qualified dieticians, and data enumerators (public health professionals) assessed the eligibility criteria of people with type 2 diabetes visiting the outpatient department of the hospital. Eligible participants were diagnosed case of T2D with HbA1c >48 mmol/mol (>6.5%), people aged ≥24−64 years, and agreed to scheduled follow-up visits. Participants were excluded if they were pregnant, lactating, planning pregnancy during the study, severely ill, had more than two comorbidities, had plans to migrate from the study area, HbA1c ≥93 mmol/mol (HbA1c ≥10.5%), and who were under insulin therapy. The study was conducted following Consolidated Standards of Reporting Trials (CONSORT) 2010 updated guidelines for reporting for randomised controlled trials, Intervention Description and Replication (TIDieR) guidelines, and local regulations. The ethical approval was obtained from the Ethical Review Board (ERB) of the Nepal Health Research Council (NHRC) (Reference # 206) and the Institutional Review Committee (IRC) from the Institute of Medicine, Tribhuvan University Teaching Hospital (Reference # 471(6−11)2 2078/2079). Written informed consent was obtained from each participant before enrolment in the study.

### Randomisation and masking

The study team members explained the purpose of the study to the participants. Those participants who were willing to give written informed consent and met the inclusion criteria were recruited to either the intervention or the usual care arm. Once a participant signed an informed written consent form, they were enrolled in the study. The trial statistician created the computer-generated random number in advance using a Microsoft Excel sheet and coded control as ‘C’ and intervention as ‘T’. In each opaque envelope, a sequential number from 1 to 156 was written outside, and the code was kept inside the envelope, which was prepared by individuals not involved in the trial. Following that, participants were assigned to the control or intervention groups based on the code assigned by the random number. To avoid bias, random numbers were kept in an envelope. The next envelope in the sequence was opened when a participant's eligibility was verified, and the intervention or control allocation was recorded on a randomisation list.

### Procedure

#### Intervention group

The nutrition education, counselling and individual diet plan were applied by trained dieticians. First, the diabetic educational manual and diet plan model were developed based on the available evidence and utilising the expertise and experiences of the health service providers, clinicians, dieticians, and people with type 2 diabetes in the management of T2D (Supplementary File). Second, a multi-stakeholder workshop was used to finalise all the intervention packages. The final design of the intervention packages was approved by multi-disciplinary experts that included an endocrinologist, dietician and other health care providers who had experience in the management of T2D. For the intervention arm, the intervention package included two phases: nutritional counselling emphasizing lifestyle modification, and an individual diet plan for each people with type 2 diabetes.

In the first phase, participants in the intervention arm received a nutrition counselling session that lasted for 30 min. The nutrition counselling package consisted of five modules: knowledge about diabetes, management of diabetes with emphasis on medical nutrition therapy, the complication of diabetes, knowledge of hypoglycaemia and its treatment, and foot care in diabetes. The modules were developed using the experiences of people with type 2 diabetes and other stakeholders who were involved in the T2D management. Nutritional counselling was provided by two trained dieticians. A follow-up was undertaken each month to reinforce the key message.

In the second phase, all people with type 2 diabetes in the intervention arm were provided with an individualised diet plan education to reinforce the concept of controlling the serving size of foods which lasted for 30 min. During the patient's visits to OPD, the dietician obtained daily nutrient intake by asking the people with type 2 diabetes to recall the foods and beverages consumed in the past 24 h. Each participant was given an individual-based diet plan within the limit of the acceptable macronutrient distribution range (AMDR).^18^ Energy distribution was set following the limit of AMDR, which comprises protein (10–35%), fat (20–35%), and carbohydrate (45–65%).^18^ The guideline was developed for the Nepalese population based on the AMDR compositions including the use of a food exchange portion in diabetic diet planning. One piece of food exchange portion is defined as one serving for every 80 kcal of a food product. Thus, a similar type of food exchange portion can be exchanged where the nutritional value is almost the same. A balanced diet for each patient was planned and the total day's diet plan is distributed into five meal patterns such as breakfast, lunch, snack, dinner, and a bedtime snack. To avoid excessive energy intake and assure a balanced diet, we emphasised the intake of moderate carbohydrates, moderate fat foods, and foods rich in fibre and micronutrients. The study participants were followed-up every month. All educational materials were developed in English and then translated into Nepali language.

#### Control group

People with type 2 diabetes in the control group (usual care arm) received routine care as to how they are practicing in their daily life. Participants in a control group could consult with a dietician from Tribhuvan University Teaching Hospital for their usual medical care and follow-up visits, but we did not provide them with the intervention package. The usual medical care included services provided by the Tribhuvan University Teaching Hospital, such as general knowledge of diabetes, pharmacological treatment, blood glucose monitoring, a healthy lifestyle, and individualised diet plan services.

### Outcomes

The primary outcome of this study was the change in HbA1c between the intervention and control groups over six months. The majority of scientists worldwide endorse using glycated haemoglobin (HbA1c) to diagnose diabetes because of its convenience and pre-analytical stability with recommended cut-off point of 6.5%.^19^

The secondary outcomes were measured in terms of a change in biochemical and clinical outcomes such as fasting blood glucose, blood pressure, lipid profile, BMI, waist circumference, Problem Areas in Diabetes (PAID) score, Diabetic Knowledge Questionnaire (DKQ), Perceived Dietary Adherence Questionnaire (PDAQ) score, and macronutrient intake. All of these were measured at baseline and end-line. Pretesting of the tools was carried out among 20 people with type 2 diabetes in Tribhuvan University Teaching Hospital before commencing the data collection. The research committee, faculty members, endocrinologists, and dieticians reviewed the pre-tested questionnaire to ensure its face validity and reliability. The questionnaires were revised appropriately based on their feedback.

The study participants’ information on socio-demographics, such as age, sex, occupation, educational level, residence, religion, and ethnicity, were collected using face-to-face interviews. The sociodemographic-related questionnaires were adapted from the Noncommunicable Disease Risk Factors: STEPS Survey Nepal 2019. To simplify the analysis, we merged similar attributes based on the observations in each cell. A fasting venous blood sample for HbA1c, fasting glucose, and lipid profile (total cholesterol, triglycerides, HDL, and LDL) were collected at baseline and at six months follow-up. All blood samples were collected, handled, analysed and stored based on the Clinical Laboratory Standards Institute (CLSI) guideline.^20^ Height was measured using a Seca 213 stadiometer. Participants' weight was measured using the Seca 874 digital weighing scale. The BMI was calculated using the formula (BMI = weight (kg)/height (m)^2^), and waist-hip circumference was measured using a measuring tape. Systolic and diastolic blood pressure (BP) was measured three times after 5 min of rest using a sphygmomanometer and stethoscope to avoid potential fluctuations due to anxiety. Here, BP measurement was also in line with a standard recommendation by The American Heart Association' (AHA') that recommends "take at least two readings and use the average of those values to represent the patient's BP".^21^

The PAID score^22^ was measured using a 20 items questionnaire measuring the problems related to emotions, treatment, food and social support. The scores for each item were summed, then multiplied by 1.25 to generate a total score out of 100. A total score of 40 or more indicated severe diabetes distress.^23^ The DKQ is a 24-items questionnaire designed by Starr County Diabetes Education Study,^24^ was used to assess the knowledge score on diabetes. There were three possible responses to the DKQ: "yes," "no," and "don't know." The correct response for each question received one point, whereas the incorrect response received no points. Each point received was summed for scoring. A higher score represents better diabetic knowledge. The PDAQ score,^25^ a 7-point Likert scale-based tool, was used to measure dietary adherence. It has a total of nine questions, with scores ranging from lowest 0 to highest 7. To get the final PDAQ score, the answers to items 4 and 9 were first reversed, and then all the answers were summed. A total score of PDAQ is 63. A higher score indicates good adherence, except for items 4 and 9, which show unhealthy choices (foods high in sugar or fat). The Cronbach’s alpha of PAID, DKQ, and PDAQ scores had found acceptable consistency of 0.83, 0.76, and 0.74, respectively.

A pre-tested 24-h dietary recall tool,^26^ based on Nepalese food and beverage, was used to assess the previous 24 h (midnight to midnight) nutrient intake of the people with type 2 diabetes. During 24-h recall, the participants were asked to name all the food and drink items consumed during the preceding day, including anything consumed outside the home and the time of consumption was also recorded. The nutrient quantification of food consumed by participants was performed using the NutriSurvey application by loading the food composition table of Nepal and neighbouring country India.

The physical activity was measured using the Global Physical activity questionnaire (GPAQ) recommended by WHO STEPwise surveillance tools.^27^ A total of 16 questions (P1–P16) assess sedentary behaviour and physical activity in three domains: work, transport, and leisure time. For each of the three domains of physical activity in the GPAQ, responses to questions on frequency and duration are used to convert total time spent on physical activity into metabolic equivalents of task (MET)-minutes. According to WHO recommendations, total physical activity is calculated as the sum of moderate-to vigorous-intensity activities performed at work, during transportation, and during leisure time. Total physical activity of ≥600 MET-minutes per week signifies meeting WHO recommendations on physical activity, while not meeting recommendations is indicated by <600 MET-minutes per week.^27^

### Statistical analysis

Since there was no similar research carried out in Nepal, the sample size was calculated based on the similar study conducted by JW Muchiri et al. (2010) in South Africa.^28^ Considering a mean difference in HbA1c (%) of 0.64 between the control group and the intervention group at six months, with a mean (SE) value of 9.67 (0.29)% in the intervention group and 10.30 (0.29)% in the control group,^28^ the sample size was calculated with a 90% power at the 5% level of significance, 54 participants per group were required. To detect a 20% difference in non-response rate, and an additional 25% was added to the sample size to compensate for the loss to follow-up, yielding 156 participants in total (78 participants in the intervention and 78 participants in the control group, respectively).

The collected data were entered in Epi-Data version 3.2 and analysed using Stata/MP version 14.1 (StataCorp LP, College Station, Texas). Baseline characteristics between the two groups were compared using the Student’s two-sample *t* test for continuous variables and the Chi-squared test for categorical variables. Data were analysed using the intention-to-treat principle. Complete case analysis was also performed excluding the seven participants (lost to follow-up = 5 and discontinued the study = 2) in the intervention group and a total of 13 participants (lost to follow-up = 9 and discontinued the study = 4) in the control group. The difference between the intervention and the control group was applied using the Student’s two-sample *t* test assuming equal variance. A linear regression analysis was used to model the primary and secondary outcome measures. We used the Multiple Imputation by Chained Equations (MICE) technique to impute the missing data for the primary and secondary outcomes and created 10 imputed datasets (seed of 53421). Multiple imputations have been demonstrated to be a reliable general approach to handling missing data in randomised clinical trial.^29^ The imputation models were the same as the analytical models. Analyses were adjusted for age, gender, place of residence, monthly family income, family history of diabetes, occupation, and physical activity (adjustments were not pre-specified and were done after knowing the imbalance between intervention and control groups with respect to these variables). Additionally, findings from minimally adjusted models (adjusted for baseline data of respective outcome variables) and complete cases are reported. All p values less than 0.05 were considered statistically significant. This trial is registered with ClinicalTrials.gov, number NCT04267367.

### Role of the funding source

The funders had no role in study design, participant selection, data collection, and data analyses. The corresponding author had full access to the data in the study and is fully responsible for the decision to submit it for publication.

## Results

**Fig. 1:**
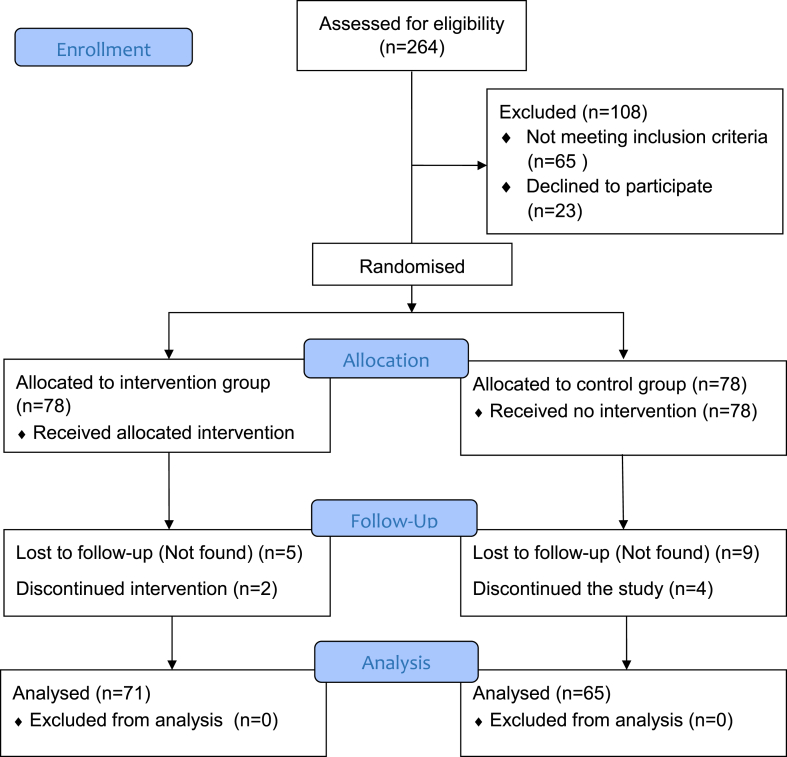
**Trialprofile.**

Between August 15, 2021 and February 25, 2022, 156 people with type 2 diabetes were recruited and randomly assigned to the intervention group (n = 78) and control group (n = 78). After six months, a total of 136 participants completed the trial, the remaining 20 people were lost to follow-up (Fig. 1).

Table 1 shows the baseline information of the study participants, which entails sociodemographic information, clinical parameters, and nutritional information. The mean (SD) age of study participants was 50.3 (9.7) years in the control group and 46.7 (10.6) years in the intervention group. At baseline, the mean (SD) HbA1c level was 8.02 (1.11)% in the control group and 8.04 (1.08)% in the intervention group, mean (SD) fasting blood glucose level was 158.12 (44.01) mg/dL in the control group and 159.15 (57.85) mg/dL in the intervention group. There were no differences in characteristics, including biochemical and clinical parameters between intervention and control groups at baseline except for gender, education, smoking and tobacco consumption, and physical activity (Table 1).

**Table 1:** Baseline characteristics of the study participants.

Variables	Control group (n = 78) n (%)	Intervention group (n = 78) n (%)
**Socio-demographic characteristics**		
Age, mean (SD) in year	50.3 (9.7)	46.7 (10.6)
**Age category**		
24–49	33 (42.3)	43 (55.1)
50–65	45 (57.7)	35 (44.9)
**Sex**		
Male	34 (43.6)	47 (60.3)
Female	44 (56.4)	31 (39.7)
**Education**		
No formal education	28 (35.9)	15 (19.2)
Formal education	50 (64.1)	63 (80.7)
**Ethnicity**		
Advantaged ethnic group	46 (58.9)	35 (44.8)
Disadvantaged ethnic group	32 (41.1)	43 (55.2)
**Religion**		
Hindu	72 (92.3)	71 (91.1)
Non-Hindu	6 (7.7)	7 (8.9)
**Marital status**		
Unmarried	3 (3.8)	2 (2.6)
Married	75 (96.2)	76 (97.4)
**Occupation**		
Employed	21 (26.9)	39 (50)
Homemaker	27 (34.6)	19 (24.4)
Unemployed	17 (21.8)	9 (11.5)
Others	13 (16.7)	11 (14.1)
**Monthly income (NPR) (1 USD = 120 Nepalese rupee)**		
≤25,000	46 (58.9)	34 (43.6)
>25,000	32 (41.1)	44 (56.4)
**Place of residence**		
Urban	23 (29.5)	27 (34.6)
Rural	55 (70.5)	51 (65.4)
**Medical history**		
**Family history of diabetes**		
Yes	34 (43.6)	30 (38.5)
No	44 (56.4)	48 (61.5)
**Behavioural characteristics**		
**Current smoking**		
Yes	7 (8.9)	19 (24.4)
No	71 (91.1)	59 (75.6)
**History of use of tobacco**		
Yes	12 (15.4)	30 (38.5)
No	66 (84.6)	48 (61.5)
**Alcohol consumption**		
Yes	9 (11.5)	33 (42.3)
No	69 (88.5)	45 (57.7)
**Clinical outcomes, mean (SD)**		
Systolic blood pressure (mm Hg)	126.08 (15.55)	128.71 (17.91)
Diastolic blood pressure (mm Hg)	83.56 (9.34)	83.41 (9.29)
BMI (kg/m^2^)	25.26 (2.69)	25.98 (5.05)
Waist circumference (cm)	84.94 (8.63)	85.93 (9.03)
Hip circumference (cm)	86.98 (7.15)	87.89 (7.31)
HbA1c (%)	8.02 (1.11)	8.04 (1.08)
FBS (mg/dL)	158.12 (44.01)	159.15 (57.85)
Total cholesterol (mg/dL)	155.79 (41.63)	156.28 (39.49)
Triglycerides (mg/dL)	155.48 (51.93)	160.87 (50.33)
LDL (mg/dL)	113.06 (35.13)	112.46 (32.04)
HDL (gm/dL)	44.97 (9.63)	43.47 (8.46)
PAID score	42.40 (18.83)	48.95 (17.39)
Diabetes knowledge score	14.92 (5.12)	14.28 (3.73)
Dietary adherence score	33.58 (8.54)	34.35 (9.12)
**WHO recommendation for physical activity, n (%)**		
Meet recommendation	50 (64.1)	52 (66.7)
Not meeting recommendation	28 (35.9)	26 (33.3)
**Nutrient intake**		
Energy (kcal/day), mean (SD)	1894.88 (337.97)	2053.93 (318.88)
**Carbohydrate**		
gm/day, mean (SD)	318.89 (81.62)	360.05 (74.84)
Energy %, mean (SD)	66.56 (6.52)	69.62 (5.25)
**Protein**		
gm/day, mean (SD)	52.13 (8.96)	53.27 (6.77)
Energy %, mean (SD)	11.28 (2.53)	10.57 (1.85)
**Fat**		
gm/day, mean (SD)	41.89 (10.14)	40.62 (9.73)
Energy %, mean (SD)	20.33 (5.40)	18.06 (4.41)
**Fibre**		
gm/day, mean (SD)	16.86 (4.01)	17.48 (4.35)
Energy %, mean (SD)	1.81 (0.48)	1.72 (0.45)

PAID: problems areas in diabetes scale.

At baseline, the total calorie and carbohydrate intake were higher in the intervention group compared to the control group, while protein, fat, and fibre consumptions were similar in both groups (Table 1).

### Primary outcomes

**Fig. 2:**
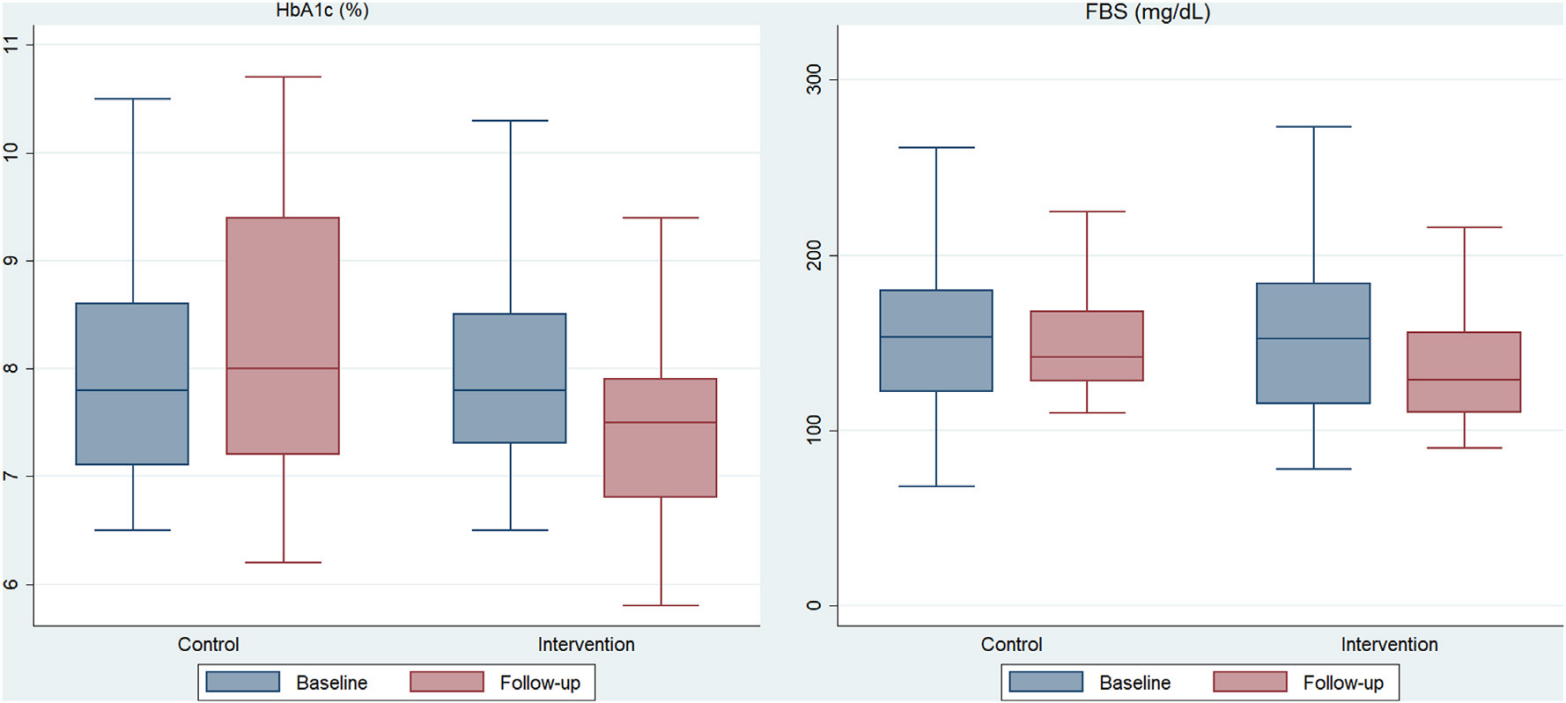
**Changes in HbA1c and fasting blood glucose (FBG) levels from baseline to follow-up in intervention and controlgroup.**

At six months, the mean HbA1c (%) level in the intervention group reduced by 0.48 (95% CI: −0.80 to −0.16), while it increased by 0.22 (95% CI: −0.21 to 0.66) in the control group (Fig. 2, Table 2). In the adjusted model, the mean reduction was 0.61 with the intervention effect size (95% CI: −1.04 to −0.17; p = 0.006) (Table 3). A similar finding was found in the multivariable model without imputation analysis (Supplementary Table S1).

**Table 2:** Mean changes in primary and secondary outcome measures from baseline to six months.

Variables	Control group (n = 65) Mean (SD)	Intervention group (n = 71) Mean (SD)	p value^a^
**Clinical outcomes**			
**HbA1c (%)**			
At baseline	7.98 (1.10)	8.02 (1.04)	0.523
At six months	8.21 (1.30)	7.53 (0.96)	0.001^b^
Change	0.22 (1.77)	−0.48 (1.36)	0.010^b^
**Fasting blood glucose (mg/dL)**			
At baseline	159.12 (44.37)	156.09 (58.73)	0.549
At six months	159.00 (46.60)	138.85 (41.40)	0.004^b^
Change	−0.12 (59.82)	−17.23 (68.95)	0.139
**Total cholesterol (mg/dL)**			
At baseline	152.29 (41.67)	156.11 (37.45)	0.529
At six months	154.24 (37.00)	147.26 (36.43)	0.135
Change	1.95 (52.63)	−8.84 (49.24)	0.549
**Triglycerides (mg/dL)**			
At baseline	154.10 (48.10)	157.59 (26.68)	0.744
At six months	149.32 (35.21)	138.81 (26.68)	0.025^b^
Change	−4.78 (59.65)	−18.77 (56.19)	0.021^b^
**LDL (mg/dL)**			
At baseline	113.09 (34.47)	113.83 (31.11)	0.455
At six months	115.93 (32.05)	110.77 (31.59)	0.173
Change	2.84 (43.84)	−3.05 (47.04)	
**HDL (mg/dL)**			
At baseline	44.29 (8.68)	43.74 (8.34)	0.151
At six months	44.53 (7.77)	45.50 (6.70)	0.781
Change	0.24 (12.15)	1.76 (11.71)	0.761
**Systolic blood pressure, mm Hg**			
At baseline	126.38 (16.14)	128.80 (17.94)	0.835
At six months	127.01 (17.53)	125.88 (12.32)	0.331
Change	0.63 (24.67)	−3.05 (22.35)	0.063
**Diastolic blood pressure, mm Hg**			
At baseline	83.66 (9.72)	83.60 (9.36)	0.459
At six months	84.07 (9.72)	82.12 (7.10)	0.091
Change	0.41 (12.63)	−1.47 (11.93)	0.346
**BMI, kg/m**^**2**^			
At baseline	25.05 (4.45)	26.04 (5.03)	0.866
At six months	25.11 (2.80)	25.69 (4.97)	0.824
Change	0.05 (0.41)	−0.35 (0.58)	0.001^b^
**Waist circumference, cm**			
At baseline	85.41 (8.79)	85.90 (8.69)	0.756
At six months	85.53 (8.58)	85.47 (8.35)	0.483
Change	0.12 (0.48)	−0.42 (0.90)	<0.001^b^
**Hip circumference, cm**			
At baseline	87.20 (6.92)	87.52 (7.04)	0.783
At six months	87.32 (6.81)	87.45 (6.91)	0.543
Change	0.12 (0.78)	−0.07 (0.48)	0.034^b^
**Problems areas in diabetes (PAID) scale**			
At baseline	43.03 (19.72)	49.41 (17.25)	0.987
At six months	44.17 (15.07)	35.88 (13.19)	0.001
Change	1.13 (25.70)	−13.53 (20.36)	<0.001^b^
**Diabetes knowledge score**			
At baseline	14.83 (5.26)	14.39 (3.78)	0.186
At six months	16.78 (3.46)	17.19 (3.91)	0.741
Change	1.95 (5.46)	2.80 (2.62)	0.132
**Dietary outcomes**			
**Dietary adherence score**			
At baseline	33.58 (8.54)	34.35 (9.12)	0.706
At six months	39.41 (8.77)	44.65 (6.62)	0.001^b^
Change	5.82 (9.66)	10.29 (9.12)	0.142
**Energy (kcal/day)**			
At baseline	1882.68 (344.64)	2060.41 (320.42)	0.998
At six months	2049.82 (397.78)	1858.29 (215.19)	0.001^b^
Change	167.13 (533.64)	−202.12 (338.04)	<0.001^b^
**Carbohydrate (gm/day)**			
At baseline	315.17 (83.02)	361.04 (75.39)	0.994
At six months	352.12 (90.87)	291.82 (50.06)	<0.001^b^
Change	36.94 (120.65)	−69.22 (80.64)	<0.001^b^
**Carbohydrate (%)**			
At baseline	66.16 (6.47)	69.58 (5.39)	0.992
At six months	67.92 (6.13)	62.58 (4.92)	<0.001^b^
Change	1.75 (8.72)	−7.00 (7.00)	<0.001^b^
**Protein (gm/day)**			
At baseline	53.61 (8.95)	53.30 (7.01)	0.814
At six months	52.22 (8.41)	57.69 (8.65)	0.007^b^
Change	−1.38 (13.19)	4.38 (12.63)	0.174
**Protein (%), mean (SD)**			
At baseline	11.38 (2.50)	10.54 (1.83)	0.025
At six months	10.80 (2.61)	12.52 (2.09)	<0.001^b^
Change	−0.58 (3.76)	1.98 (2.80)	<0.001^b^
**Fat (gm/day)**			
At baseline	42.10 (9.81)	40.83 (9.97)	0.213
At six months	43.43 (10.58)	46.61 (8.93)	0.059
Change	1.32 (15.31)	5.78 (13.12)	0.023^b^
**Fat (%)**			
At baseline	20.58 (5.35)	18.11 (4.53)	0.002
At six months	19.45 (4.81)	22.68 (4.01)	<0.001^b^
Change	−1.12 (7.27)	4.56 (6.01)	<0.001^b^
**Fibre (gm/day)**			
At baseline	17.04 (4.14)	17.75 (4.24)	0.821
At six months	17.98 (3.91)	20.32 (4.22)	0.001^b^
Change	0.93 (5.84)	2.56 (5.89)	0.068
**Fibre (%)**			
At baseline	1.85 (0.51)	1.75 (0.43)	0.117
At six months	1.82 (0.50)	2.20 (0.49)	<0.001^b^
Change	−0.04 (0.65)	0.45 (0.66)	<0.001^b^

a Student’s two-sample *t* test.

b Statistically significant at p < 0.05.

**Table 3:** Changes in primary and secondary outcomes after six months intervention.^e^

Variables	Unadjusted model		Adjusted model	
	Intervention effect size (95% CI)^a^	p value^b^	Intervention effect size (95% CI)^a^	p value^c^
**Clinical outcomes**				
HbA1c (%)	−0.69 (−1.10 to −0.28)	0.001^d^	−0.61 (−1.04 to −0.17)	0.006^d^
Fasting blood glucose (mg/dL)	−19.12 (−34.93 to −3.32)	0.018^d^	−18.96 (−36.12 to −1.81)	0.031^d^
Total cholesterol (mg/dL)	−8.52 (−20.82 to 3.77)	0.173	−9.92 (−22.34 to 2.48)	0.116
Triglycerides (mg/dL)	−10.55 (−20.82 to 0.27)	0.044^d^	−11.93 (−22.96 to 0.90)	0.034^d^
LDL (mg/dL)	−5.09 (−15.61 to 5.42)	0.340	−6.66 (−17.39 to 4.07)	0.222
HDL (mg/dL)	1.18 (−1.32 to 3.68)	0.351	0.46 (−2.15 to 3.08)	0.726
Systolic blood pressure (mm Hg)	−1.06 (−6.80 to 4.67)	0.711	−1.59 (−7.58 to 4.39)	0.596
Diastolic blood pressure (mm Hg)	−1.92 (−4.84 to 0.98)	0.192	−2.30 (−5.38 to 0.77)	0.141
BMI (kg/m^2^)	−0.39 (−0.56 to −0.22)	<0.001^d^	−0.36 (−0.55 to −0.17)	<0.001^d^
Waist circumference (cm)	−0.54 (−0.77 to −0.31)	<0.001^d^	−0.52 (−0.77 to −0.27)	<0.001^d^
Hip circumference (cm)	−0.18 (−0.39 to 0.03)	0.093	−0.25 (−0.47 to −0.03)	0.025^d^
Problems areas in diabetes (PAID) scale	−8.06 (−12.87 to −3.25)	0.001^d^	−8.22 (−13.34 to −3.10)	0.002^d^
Diabetes knowledge score	0.55 (−0.57 to 1.68)	0.333	0.52 (−0.68 to 1.72)	0.394
**Dietary outcomes**				
Dietary adherence score	4.87 (2.31–7.43)	<0.001^d^	5.24 (2.56–7.93)	<0.001^d^
Energy (kcal/day)	−203.27 (−314.45 to −91.99)	0.001^d^	−231.84 (−351.50 to −112.19)	<0.001^d^
Carbohydrate (gm/day)	−61.45 (−86.78 to −36.12)	<0.001^d^	−70.91 (−97.88 to −43.94)	<0.001^d^
Carbohydrate (%)	−5.53 (−7.49 to −3.57)	<0.001^d^	−6.14 (−8.17 to −4.11)	<0.001^d^
Protein (gm/day)	4.22 (1.35–7.10)	0.004^d^	3.75 (0.77–6.73)	0.014^d^
Protein (%)	1.67 (0.85–2.49)	<0.001^d^	1.76 (0.87–2.65)	<0.001^d^
Fat (gm/day)	2.63 (−0.67 to 5.93)	0.118	3.81 (−0.37 to 7.25)	0.030^d^
Fat (%)	3.21 (1.66–4.76)	<0.001^d^	3.80 (2.23–5.36)	<0.001^d^
Fibre (gm/day)	2.58 (0.94–4.22)	0.003^d^	2.26 (0.56–3.95)	0.010^d^
Fibre (%)	0.40 (0.23–0.57)	<0.001^d^	0.40 (0.22–0.58)	<0.001^d^

a Intervention effect size and 95% CIs calculated using linear regression model.

b p-value for unadjusted model.

c p-value for adjusted model after adjusted for age, gender, place of residence, monthly family income, family history of diabetes, occupation, and physical activity.

d Statistically significant at p < 0.05.

e Intention-to-treat analysis.

### Secondary outcomes

At six months of follow-up, the mean fasting blood glucose level was reduced by 19.38 mg/dL in the intervention group, whereas it decreased by 0.12 mg/dL in the control group (Fig. 2, Table 2). The dietary intervention was associated with a mean reduction of 18.96 mg/dL (95% CI: −36.12 to −1.81; p = 0.031) in the adjusted model. There was no statistically significant difference in total cholesterol, LDL-cholesterol, and HDL-cholesterol between the intervention and control groups. In the adjusted model, the mean reduction in triglycerides was 11.93 mg/dL (95% CI: −22.96 to 0.90; p = 0.034) (Table 3). The intervention group had a 0.36 kg/m^2^ (95% CI: −0.55 to −0.17; p < 0.001) reduction in BMI compared to usual care. Similarly, the waist and hip circumferences were reduced by 0.52 cm (95% CI: −0.77 to −0.27; p < 0.001), and 0.25 cm (95% CI: −0.47 to −0.03; p = 0.025), respectively. The intervention resulted in a mean reduction of Problems Areas in Diabetes (PAID) score of 8.22 (95% CI: −13.34 to −3.10; p = 0.002) (Table 3). These findings were similar to multivariable analysis without imputation analysis (Supplementary Table S1).

At six months of follow-up, the dietary adherence score was increased by 5.24 (95% CI: 2.56–7.93; p < 0.001). Similarly, the dietary intervention was associated with a mean calorie and carbohydrate reduction of −231.84 kcal (95% CI: −351.48 to −112.21; p < 0.001) and −70.91 g (95% CI: −97.88 to −43.94; p < 0.001), respectively. The mean percentage of carbohydrate intake also decreased by 6.14% (95% CI: −8.17 to −4.11; p < 0.001) and reached within the AMDR limit in the intervention group. The consumption of protein, fat, and fibre significantly increased in the intervention group and the percentage of these nutrient intakes attained the AMDR limit (Tables 2 and 3). At six months of follow-up, the mean (SD) the percentage of protein, and fat intake were 12.52% (2.09), and 22.68% (4.01), respectively (Table 2).

## Discussion

The current study demonstrated that the dietician-led dietary intervention was found effective in reducing HbA1c levels, different biochemical and clinical parameters, and improving macronutrient intake among people with type 2 diabetes. This study highlights the roles and necessity of dieticians in the management of diabetes in all public and private hospitals in LMICs such as Nepal. If a dietician is involved in the care of diabetes through dietary approaches along with a clinician, it will have a higher impact on lowering blood glucose levels and promoting dietary habits.

The findings of this research demonstrated that diet plans delivered by dieticians and diet counselling interventions were effective in reducing HbA1c (%) by 0.48 and fasting blood glucose levels by 18.96 mg/dL in people with type 2 diabetes. These results are in line with the findings from studies conducted in Taiwan (HbA1c; −0.5%),^16^ Italy (HbA1c; −0.4%),^15^ and South Africa (HbA1c; −0.63%)^28^ that showed nutrition education was effective in lowering HbA1c levels. The benefit of offering nutrition counselling by a dietician to people with type 2 diabetes has recently been highlighted by several studies.^30^^,^^31^ In most dietician-led dietary counselling interventions, the role of the dietician is to provide counselling on dietary habits, portion sizes, and a healthy diet. Evidence suggests that nutrition education and counselling could have a greater impact on improving glycaemic control (lower mean HbA1c and fasting plasma glucose), and minimising complications associated with diabetes. The plausible physiological mechanism could be that diets stimulate a variety of metabolic processes and alter the body's metabolic integrity.^32^ As a result, unhealthy eating patterns have been one of the main causes of glucose metabolism disorder that eventually leads to the development of diabetes.^33^

We found a significant reduction in BMI and waist circumference in the intervention group, as opposed to an increase in the control group. This result corroborates the findings from the research conducted in South Africa, where BMI was significantly lower in the intervention group than in the control group.^28^ Furthermore, some studies have reported a slight improvement in BMI.^15^^,^^16^ Nutrition education broadens a person's understanding of energy balance so that they can develop the communication skills needed to describe how diets operate in accordance to help people attain and maintain healthy body weight and composition.^32^ Most commonly, T2D is linked to being overweight or obese. Losing weight has been associated with improvements in glycaemic control, BP, and cholesterol levels, which can delay or prevent complications, especially cardiovascular problems.^10^ Loss of weight is attributed to a decrease in HbA1c.^34^ Another important aspect of diabetes management is physical activity, which helps the body use insulin more efficiently.

The findings of this research demonstrated that the dietician-led intervention significantly improved the dietary adherence score and problem areas in diabetes (PAID) scale. The previous studies also showed that diabetic education could improve the PAID scale^11^ and dietary adherence.^13^ The PAID assesses the negative emotions associated with having diabetes; higher scores indicate greater distress and may help identify depressive and emotional issues in people with diabetes.^35^ Individualised nutrition education consequently improved glycaemic control and helped people adhere to diet recommendations.^13^

This study also showed that the nutrition education intervention and diet plan provided by the dietician significantly improved the macronutrient intake in the intervention group compared to the control group. Total energy and carbohydrate intake predominantly decreased in the intervention group, whereas both were observed to increase in the control group. On the other hand, the mean intake of protein, fat, and fibre consumption increased in the intervention group. Similar results were reported in the previous studies.^16^^,^^36^ People with diabetes who received diabetes nutrition education exhibited improved eating patterns and nutritional knowledge, which improved nutrient intake levels.^10^^,^^13^^,^^28^ European^37^ and Canadian guidelines on nutrition therapy recommend that,^38^ protein should make up 10–20% of total calories, carbohydrates should make up 45–60%, and fat should make up no more than 35%. Similarly, dietary guidelines for healthy living and prevention of obesity, in Asian Indians recommend obtaining 50–60% of energy from carbohydrates, 10−15% from protein, and less than 30% from fat.^39^ Despite a considerable reduction in carbohydrate intake from the baseline, the intervention group had attained an acceptable macronutrient intake level.

Our study highlighted the importance of a dietary approach for managing diabetes in low-income and middle-income countries like Nepal. Female Community Health Volunteers delivered health promotion interventions in Pokhara, Nepal, has highlighted the importance of contextual factors such as community mobilization and education of communities for the management of diabetes.^40^ Western dietary pattern has influenced the locally available traditional Nepalese diet and Nepalese people consume fewer fruits and vegetables, only two days in a week.^41^ Prioritising and scaling up dietary approaches in the existing healthcare system of Nepal with improved nutrition literacy can improve healthy eating habits among people with type 2 diabetes. This can be achieved through the integration of dietary approaches in the implementation of the Package of Essential Non-communicable Diseases (PEN) and Multi-sectoral Action Plan for Prevention and Control of NCDs in Nepal.

One of the main strengths of the study is that the intervention package was designed and delivered by a dietician. The study followed a randomised controlled study design with the control group and intervention group; and undertook monthly follow-ups to track and emphasise key messages to people with type 2 diabetes. Furthermore, all biochemical samples and anthropometric data were collected by health workers and were standardised. However, there were some inevitable limitations in the study design. First, the study could not be blinded which may have introduced biases. Second, the participants were enrolled on an out-patient department (OPD) basis, so we could not resolve the effects of dietary adherence recommended during the intervention. Third, we collected the nutrient intake level using a single 24-h dietary recall that did not include the usual food intake. Fourth, even though we included participants taking hyperglycaemic agents in both the control and intervention groups, we could not precisely record the names of the medicines. Fifth, we did not perform the per protocol analysis as the study did not collect information related to the level of adherence to the intervention or fidelity of intervention.

This study concludes that dieticians who delivered nutrition education and diet plan significantly improved HbA1c and fasting blood glucose level, reduced BMI, and waist-hip circumference, lower carbohydrate intake, and increased consumption of protein, fat and fibre. A significant improvement in glycaemic management in people with type 2 diabetes can be expected if such intervention is offered routinely in LMICs.

## Contributors

Conceptualisation: DRSu and DRSi. Data analysis and data curation: DRSu, DRSi, and RD. Data collection and entry: SN, KN, and MT. Project administration: DRSu, DRSi, SN, NS, MS, PKS, and RY. Funding acquisition: DRSu, DRSi, and PP. Software: DRSu, DRSi, and RD. Supervision: DRSu, DRSi, KK, PMSP, PP, NS, and PKS. Writing-original draft: DRSu, DRSi, SN, RD, KK, and PMSP. Writing-critical review and editing: DRSu, SN, RD, KK, PMSP, PP, KN, MT, NS, MS, PKS, RY, and DRSi. All authors read and approved the manuscript.

## Data sharing statement

All relevant data is available in the paper. De-identified dataset will be made available to researchers upon reasonable request after publication, and any further requirements would be welcomed. Requests for data should be addressed to the corresponding author.

## Declaration of interests

DRSu and DRSi received funding from University Grant Commission, Nepal under Faculty research grant support (Award number: FRG-76/77-HS-2). All authors declare no other conflicts of interests.

## Appendix A Supplementary data

Supplementary File

Study Protocol
