# Supplementary material for: Effectiveness of a dietician-led intervention in reducing glycated haemoglobin among people with type 2 diabetes in Nepal: a single centre, open-label, randomised controlled trial
Source: Lancet Reg Health Southeast Asia. 2023 Sep 25;18:100285. doi: 10.1016/j.lansea.2023.100285 (PMC10667281; doi:10.1016/j.lansea.2023.100285)

**Effectiveness of a dietician-led intervention in reducing glycated haemoglobin among people with type 2 diabetes in Nepal: a single centre, open-label, randomised controlled trial**

**Supplementary Table 1**. **Univariate and multivariable linear regression model of primary and secondary outcomes after receiving six-month interventions^#^.**

| **Variables** | **Unadjusted model** | | **Adjusted model** | |
| --- | --- | --- | --- | --- |
|  | **Intervention effect size (95% CI)^¥^** | **P value^†^** | **Intervention effect size (95% CI)^¥^** | **P value^‡^** |
| **Clinical outcomes** | | | | |
| HbA1c (%) | -0.67 (-1.06 to -0.28) | 0.001* | -0.58 (-1.00 to -0.17) | 0.006* |
| Fasting blood glucose (mg/dL) | -19.88 (-34.78 to -4.96) | 0.009* | -19.38 (-35.43 to -3.33) | 0.018* |
| Cholesterol (mg/dL) | -7.36 (-19.81 to 5.07) | 0.244 | -9.10 (-21.88 to 3.67) | 0.161 |
| Triglycerides (mg/dL) | -10.40 (-20.98 to 0.17) | 0.054 | -11.12 (-22.59 to 0.33) | 0.057 |
| LDL (mg/dL) | -5.16 (-16.01 to 5.67) | 0.348 | -6.16 (-17.35 to 5.01) | 0.277 |
| HDL (mg/dL) | 0.90 (-1.53 to 3.34) | 0.466 | 0.26 (-2.36 to 2.89) | 0.843 |
| Systolic blood pressure (mm Hg) | -0.98 (-6.11 to 4.14) | 0.705 | -1.37 (-6.94 to 4.19) | 0.626 |
| Diastolic blood pressure (mm Hg) | -1.94 (-4.82 to 0.92) | 0.183 | -2.25 (-5.40 to 0.88) | 0.157 |
| BMI (kg/m^2^) | -0.39 (-0.56 to -0.22) | <0.001* | -0.37 (-0.56 to -0.18) | <0.001* |
| Waist circumference (cm) | -0.52 (-0.75 to -0.30) | <0.001* | -0.52 (-0.76 to -0.27) | <0.001* |
| Hip circumference (cm) | -0.18 (-0.40 to 0.02) | 0.086 | -0.25 (-0.48 to -0.03) | 0.023* |
| Problems areas in diabetes (PAID) scale | -8.37 (-13.26 to -3.48) | 0.001* | -8.48 (-13.77 to -3.19) | 0.002* |
| Diabetes knowledge score | 0.58 (-0.51 to 1.68) | 0.293 | 0.50 (-0.69 to 1.70) | 0.407 |
| **Dietary outcomes** | | | | |
| Dietary adherence score | 4.87 (2.31 to 7.43) | <0.001* | 5.24 (2.56 to 7.93) | <0.001* |
| Energy (kcal/day) | -203.22 (-314.44 to -92.01) | <0.001* | -231.84 (-351.48 to -112.21) | <0.001* |
| Carbohydrate (gm/day) | -64.57 (-90.17 to -38.96) | <0.001* | -72.29 (-99.48 to -45.09) | <0.001* |
| Carbohydrate (%) | -5.53 (-7.49 to -3.57) | <0.001* | -6.14 (-8.17 to -4.11) | <0.001* |
| Protein (gm/day) | 4.35 (1.41 to 7.280) | 0.004* | 4.04 (0.91 to 7.16) | 0.012* |
| Protein (%) | 1.67 (0.85 to 2.49) | <0.001* | 1.76 (0.87 to 2.65) | <0.001* |
| Fat (gm/day) | 3.12 (-0.20 to 6.45) | 0.066 | 3.91 (0.44 to 7.38) | 0.027* |
| Fat (%) | 3.21 (1.66 to 4.76) | <0.001* | 3.80 (2.23 to 5.36) | <0.001* |
| Fibre (gm/day) | 2.34 (0.95 to 3.74) | 0.001* | 2.05 (0.58 to 3.52) | 0.006* |
| Fibre (%) | 0.40 (0.23 to 0.57) | <0.001* | 0.40 (0.22 to 0.58) | <0.001* |
| ^¥^Intervention effect size and 95% CIs calculated using linear regression model; ^†^p-value for unadjusted model; ^‡^p-value for adjusted model after adjusted for age, gender, place of residence, monthly family income, family history of diabetes, occupation and physical activity; *statistically significant if p<0.05; ^#^Complete case analysis. | | | | |

**Intervention**

The intervention is focused on the adoption of healthy eating habits that are behaviourally informed and practicable. To address the uncertainties around dietary advice, this study draws on the relationship between the dietician and the patient through diabetic education manual and diet plan. The intervention incorporated structured materials offered by the dietician to improve the glucose profile, clinical parameters, and dietary intake among type 2 diabetes (T2D) patients. The intervention package included two-phases; phase I: nutritional counselling emphasis on lifestyle modification of diabetes patients using diabetic education manual and phase II: individual basis diet plan.

1. **Diabetes Education Manual Development Process**

For the preparation of diabetic education manual, a team of nutrition and public health experts (also the research team) conducted a literature review. Literature review was conducted assessing guidelines such as Indian Council for Medical Research (ICMR) guidelines for management of type 2 diabetes^1^, International Diabetes Federation diabetes atlas^2^, and standards of medical care in diabetes by American Diabetes Association^3^. Diabetic education manual was divided into five modules (knowledge about diabetes, management of diabetes emphasis on medical nutrition therapy, the complication of diabetes, knowledge of hypoglycaemia and hyperglycaemia and its treatment, and foot care in diabetes) and task of preparing the module was designated within the multi-disciplinary expert team comprising an endocrinologist, public health expert and dietician. The draft manual was first prepared in English version and was reviewed by the team. The team exchanged the module they prepared with each other and provided comments and suggestions based on the review. This was followed by a group meeting of team members to finalise the English version draft which was then translated in Nepali language by a professional bilingual translator. The Nepali version of the draft was provided to a graphic designer to design the manual in an attractive, convenient, and easy to read approach. Various infographic mediums were used in 2D print model of diabetic education manual to make it much easier and convenient to read by the patients with diabetes. The designed and printed draft was then provided to a multidisciplinary expert that included an endocrinologist, dietician and other health care providers involved in the management of type 2 diabetes for further review.

A workshop was organized to review the draft of diabetic education manual in which 12 stakeholders participated. The infographics were presented among patients with diabetes, and their family members; endocrinologist; other health care professionals such as nurses and public health professional; academicians and dietician. The workshop was organized to collect the view from the experts and the patients to refine it. Workshop participants were provided with hand-outs of the presentation in Nepali language to ensure we had their adequate feedbacks. Also, the manual was read out line by line in the workshop and views from participants (experts and patients) were taken on aspects of perception towards the manual, comprehensibility, utility, feasibility in cultural context, and suggestions and recommendations regarding the diabetic education manual. The suggestions and corrections to the manual were incorporated to adapt it.

Some of the feedback offered us valuable insights. For instance, major revision was made on making the manual fit to the cultural context (for example: mentioning locally available food items in diet, cooking procedures etc.). This revised manual was then again presented to experts and after their final suggestions, manual was printed in required quantity for testing it through dieticians.

For testing the manual in intervention, the two trained dieticians in the study were our study team members (Chitrakala Nepal and Madhu Thapa) who have M.Sc. nutrition and dietetics degree. In addition, these dieticians were further provided with two days of intensive training to offer information on diabetes as per the manual. The training was provided by senior dietician (Nani Shobha Shakya and Dev Ram Sunuwar) to familiarise the modules of the diabetic education manual and within the specified time frame. Dieticians were pre and post tested; and practical sessions and examinations were undertaken to assess the training process and its outcome.

The qualitative interviews were conducted among a multidisciplinary team member comprising endocrinologist (n=1), dietician (n=3), academician (n=1), and patient (n=2) to test the feasibility and acceptability of the manual. The COREQ ([Consolidated criteria for reporting qualitative research](https://www.equator-network.org/reporting-guidelines/coreq/)) checklist was used to develop the interview guide and trained public health professionals were recruited to conduct qualitative interviews^4^. A natural setting was created in interview process and feasibility and acceptability of the manual was accessed, probes were introduced where needed and the collected data were coded and categorized into themes and subthemes. The collected ideas, opinions and suggestions were taken into consideration while drafting the final manual.

The final version of diabetic education manual was used for the intervention in the Randomised Control Trial (RCT). Any further suggestions and recommendations were taken from the experts and stakeholders and were incorporated in the final diabetes education manual. Dieticians were again trained according to the redesigned final diabetes education manual to execute the educating process for RCT (Annex 1: Diabetic education manual).

1. **Diet Plan Development Process**

The diet plan was developed with consideration of type of food eaten in the Nepalese cultural context. It included food for a day (five meals that comprised diet to take just after waking up, breakfast, lunch, teatime, and dinner). Quantity and type of diet to be consumed was prescribed by the dietician according to age, sex, weight, Body Mass Index (BMI), physical activity level, and type of diet (vegetarian or non-vegetarian). Each participant was given an individual basis diet plan within the limit of the acceptable macronutrient distribution range (AMDR) ^5^. Acceptable Macronutrient Distribution Range is the recommended proportion of a person’s daily calories that should come from protein, fat, and carbohydrates. The individual total energy required per day is calculated according to the individual weight and daily physical activity level using Harris-Benedict equations. Energy distribution was set in accordance with the limit of AMDR such as protein 10-35%, fat 20-35%, and carbohydrate 45-65%)^5^. This guideline was developed for the Nepalese population based on the AMDR that includes the use of a food exchange portion in diabetic diet planning. One piece of food exchange portion is defined as one serving for every 80 kcal of a food product. Thus, a similar type of food exchange portion can be exchanged where the nutritional value is almost the same.

Dieticians were trained to create the diet plan; a practical session was organized to build the capacity to correctly plan the diet plan with respect to individual patient characteristics and assessment. Dieticians were only recruited for real case scenario after they had significant progress in post-test. Calorie requirements were analysed and prescribed by the trained dietician. Estimated nutrition was categorised into protein, carbohydrate, fats in gram and percentage and cholesterol in milligram. Considering comfort and convenience of patients with diabetes, food exchange list was developed. Here, patient could choose and exchange varied type of food to eat without affecting the balanced diet and diverting from diet plan.

**References**

1 Indian Council of Medical Research AN, New Delhi – 110 029. ICMR GUIDELINES FOR MANAGEMENT OF TYPE 2 DIABETES. 2018 https://main.icmr.nic.in/sites/default/files/guidelines/ICMR_GuidelinesType2diabetes2018_0.pdf (accessed Oct 23, 2022).

2 International Diabetes Federation. IDF Diabetes Atlas | Tenth Edition. 2021. https://diabetesatlas.org/ (accessed Oct 5, 2022).

3 American Diabetes Association. Standards of Medical Care in Diabetes—2022 Abridged for Primary Care Providers. *Clin Diabetes* 2022; **40**: 10–38.

4 Tong A, Sainsbury P, Craig J. Consolidated criteria for reporting qualitative research (COREQ): a 32-item checklist for interviews and focus groups. *Int J Qual Heal Care* 2007; **19**: 349–57.

5 Institute of Medicine. Dietary Reference Intakes for Energy, Carbohydrate, Fiber, Fat, Fatty Acids, Cholesterol, Protein, and Amino Acids. *Diet Ref Intakes Energy, Carbohydrate, Fiber, Fat, Fat Acids, Cholesterol, Protein, Amin Acids* 2002; : 1–1331.

# **Annex 1**

# **Diabetic education manual (in Nepali)**


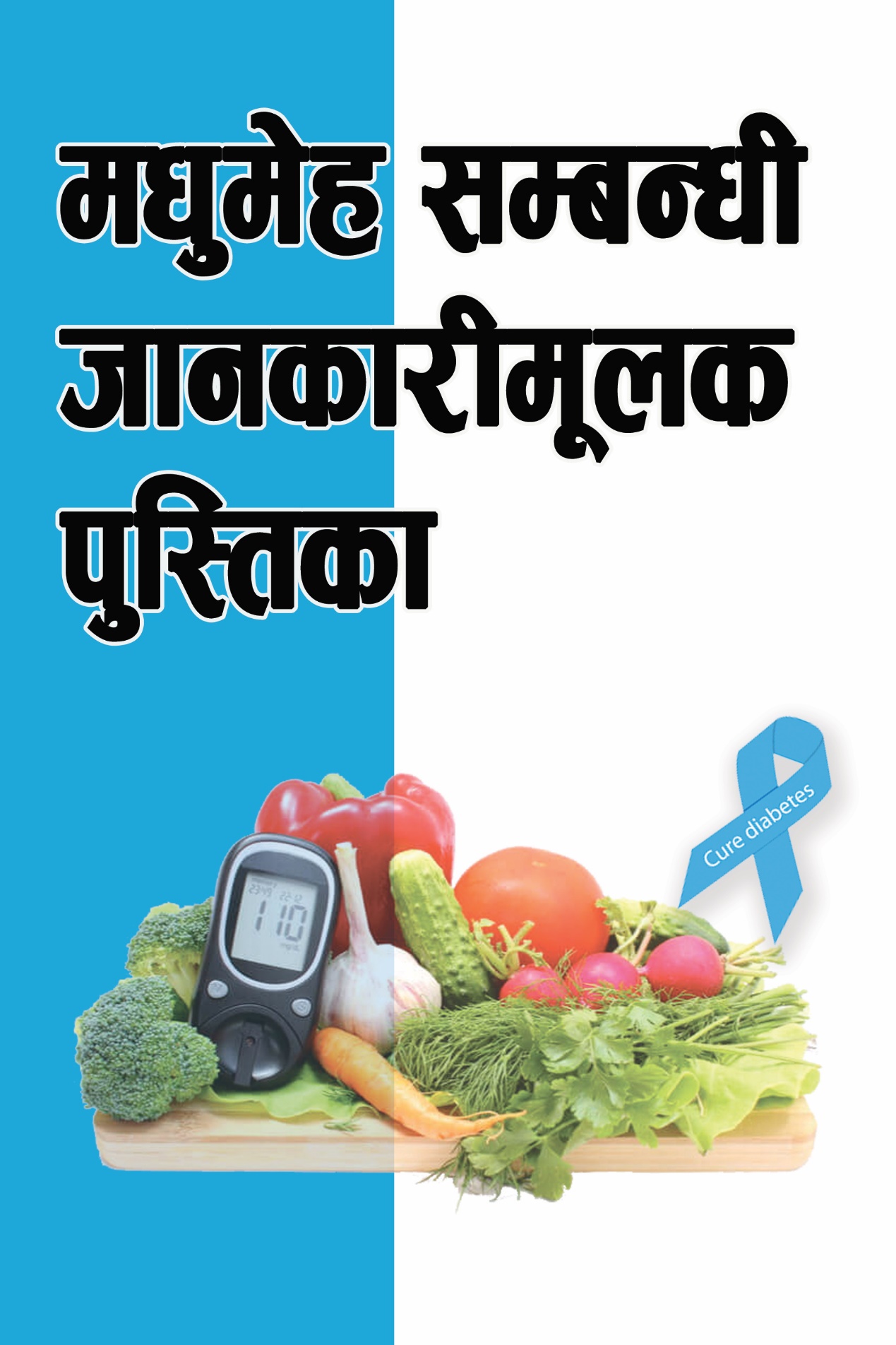


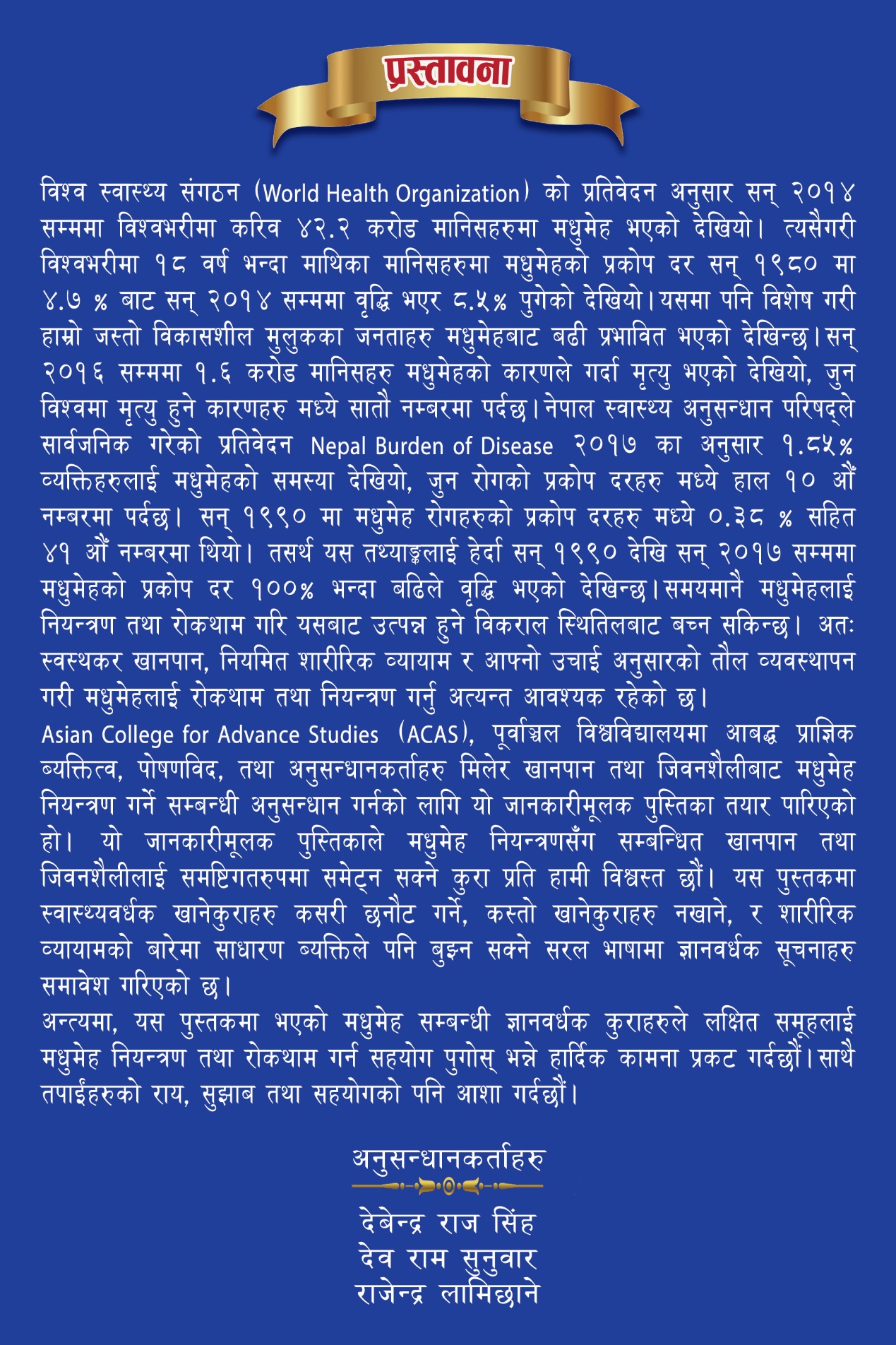


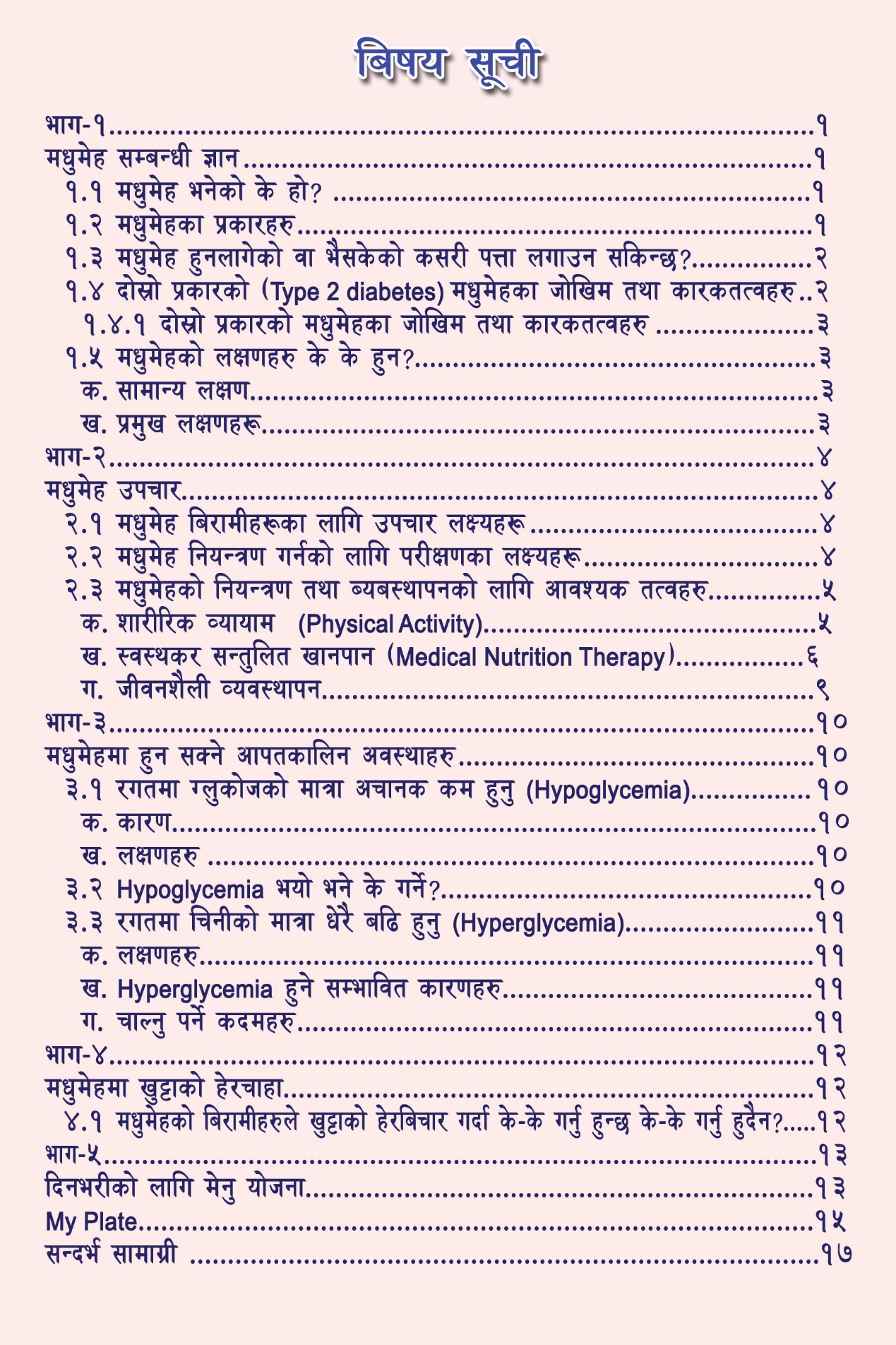


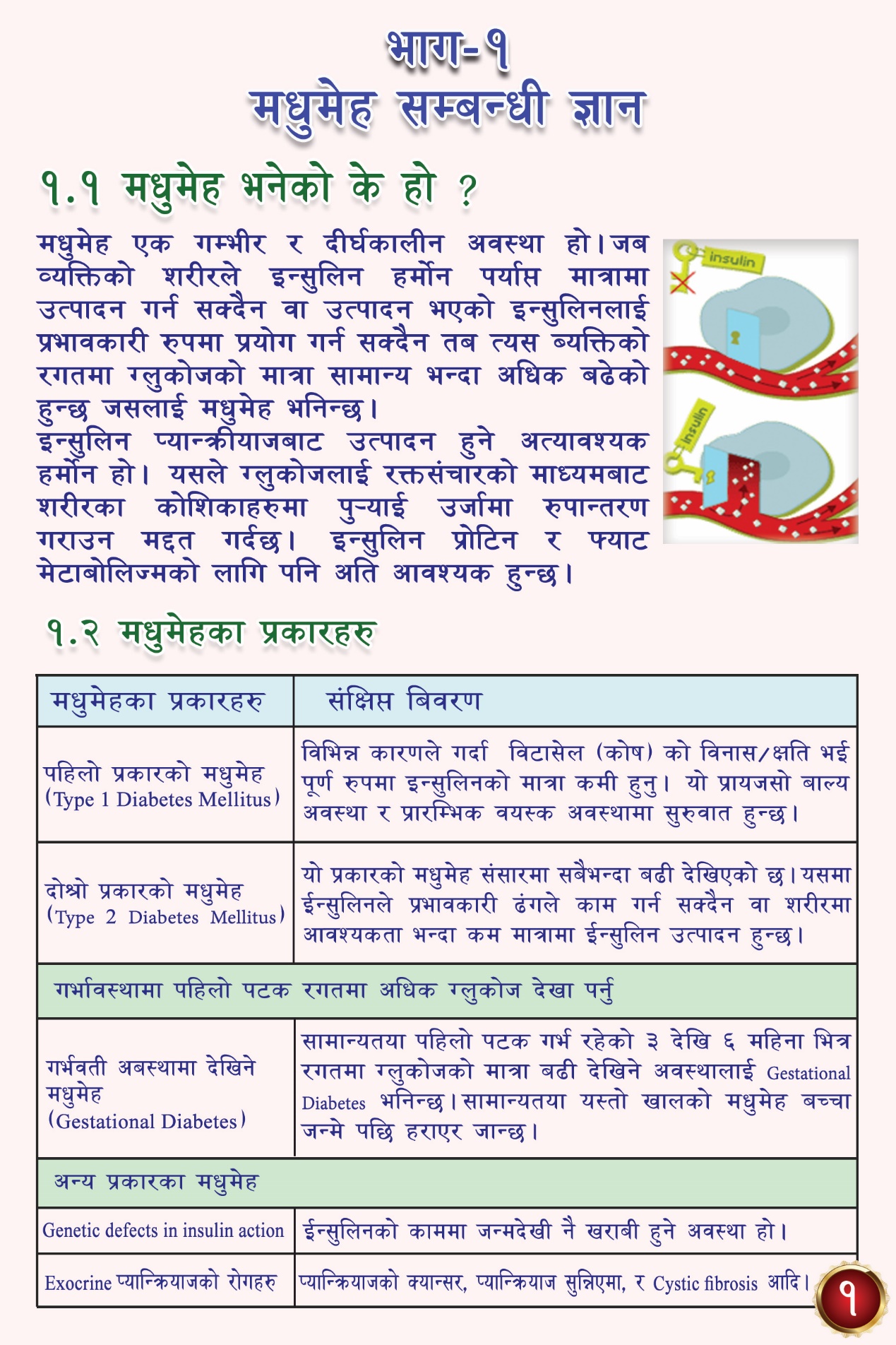


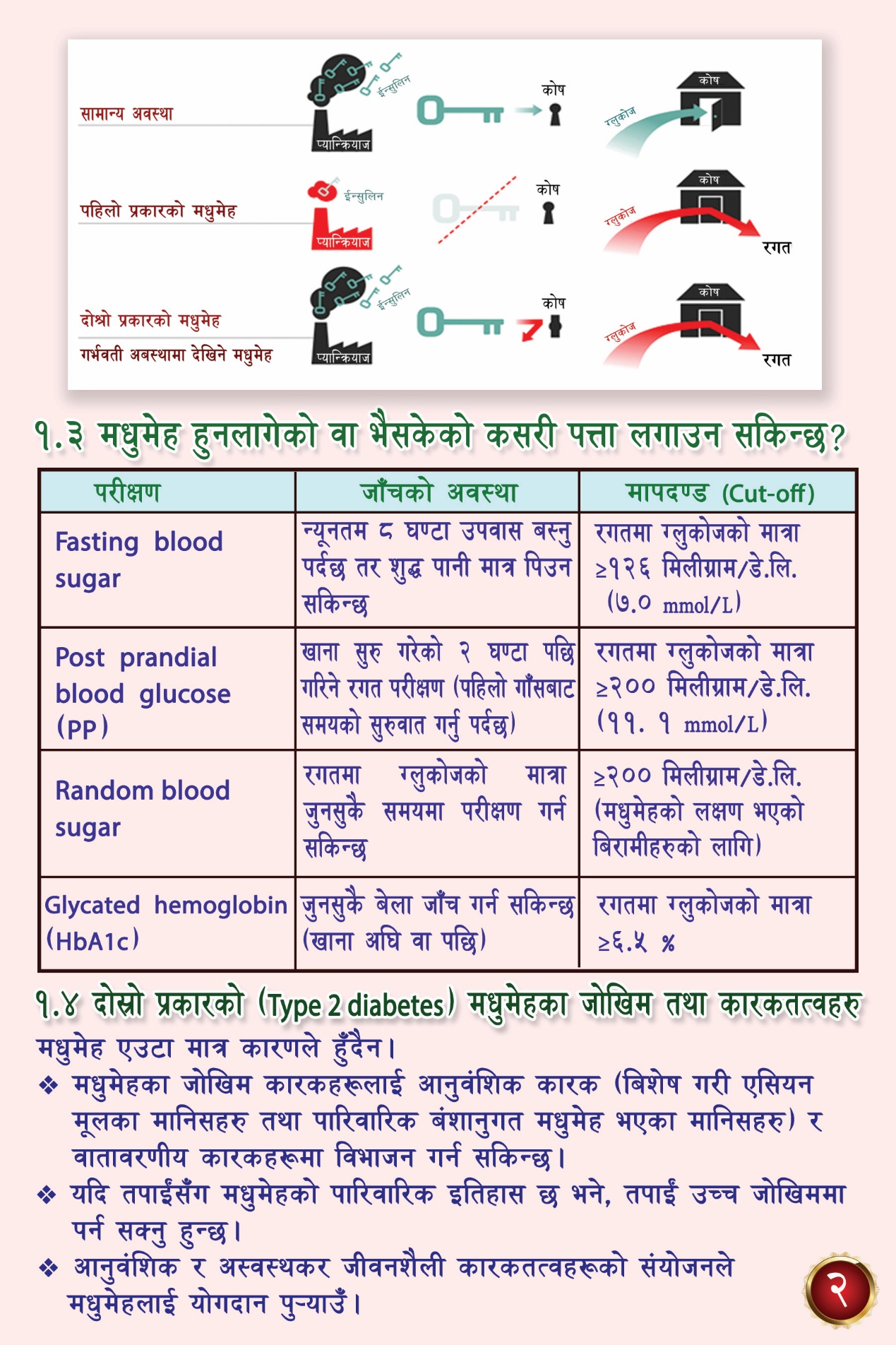


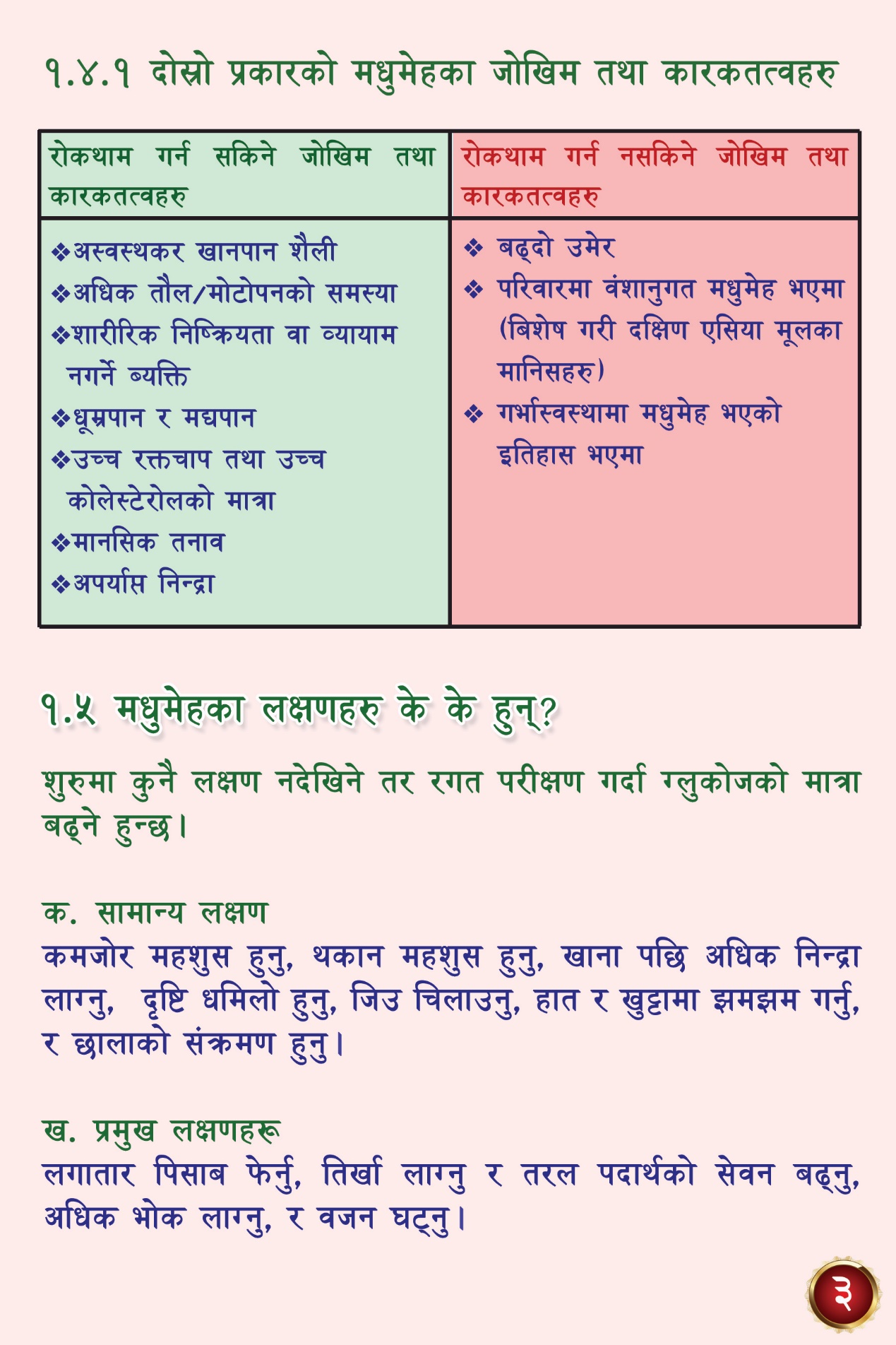


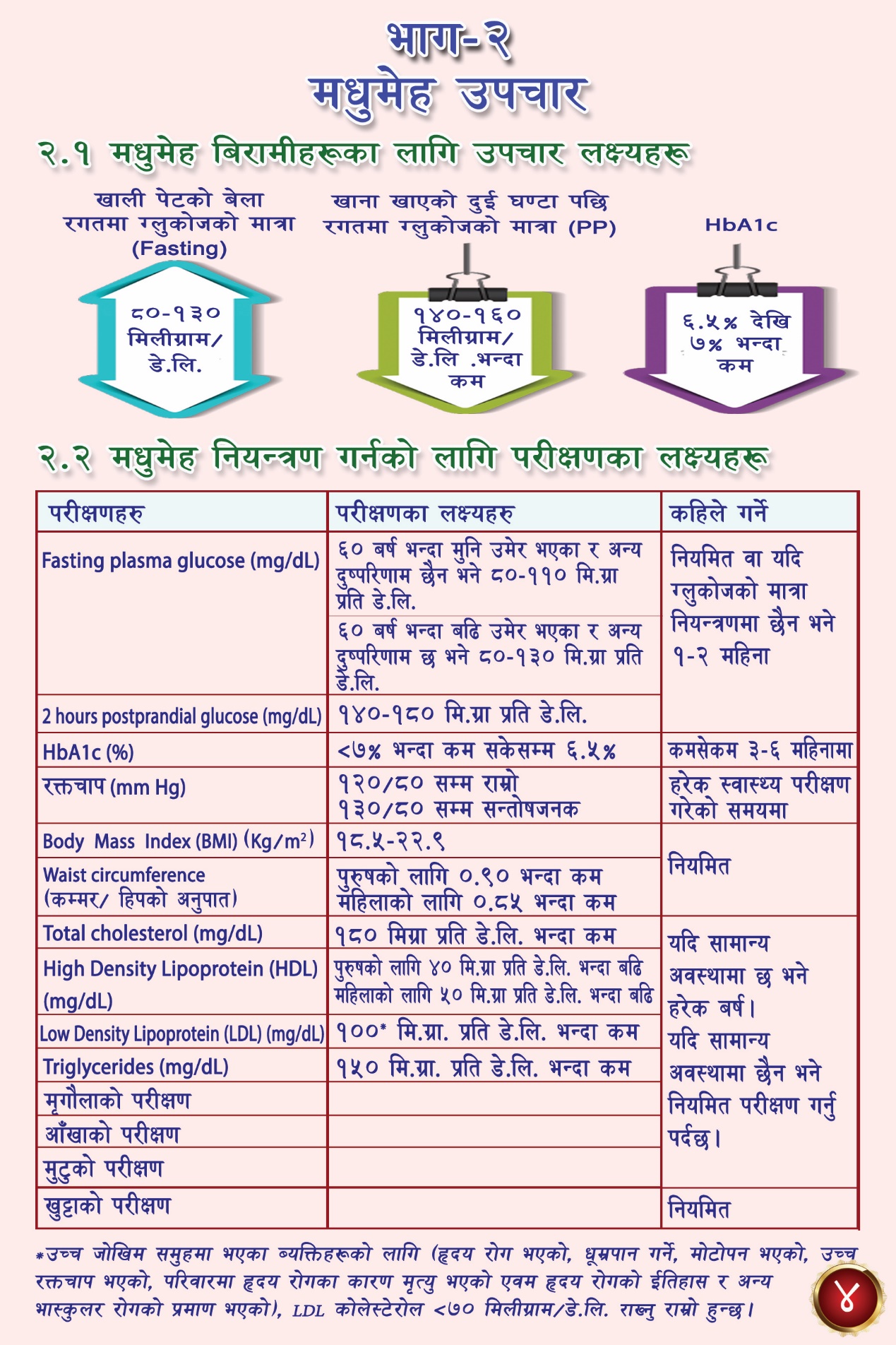


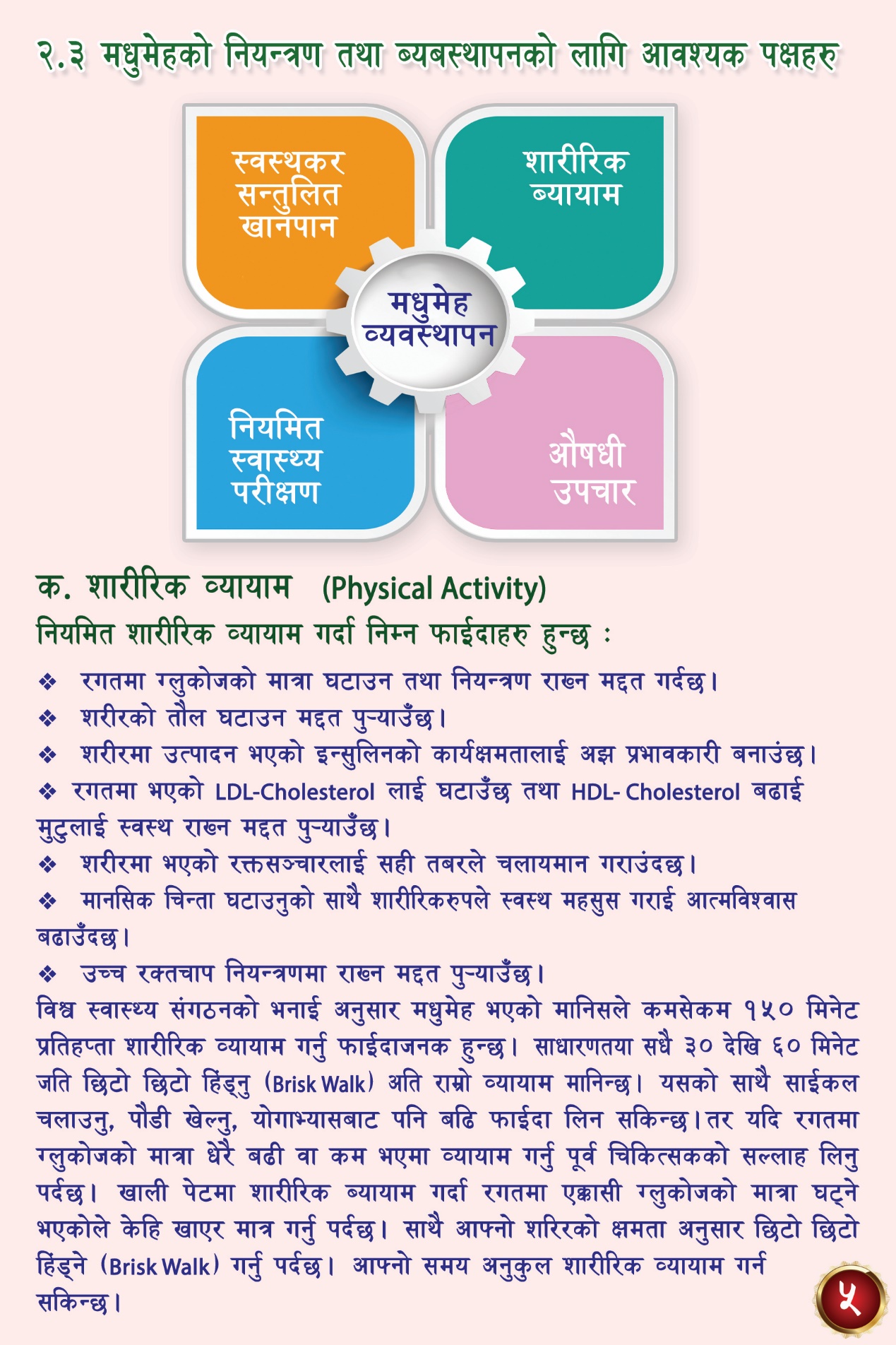


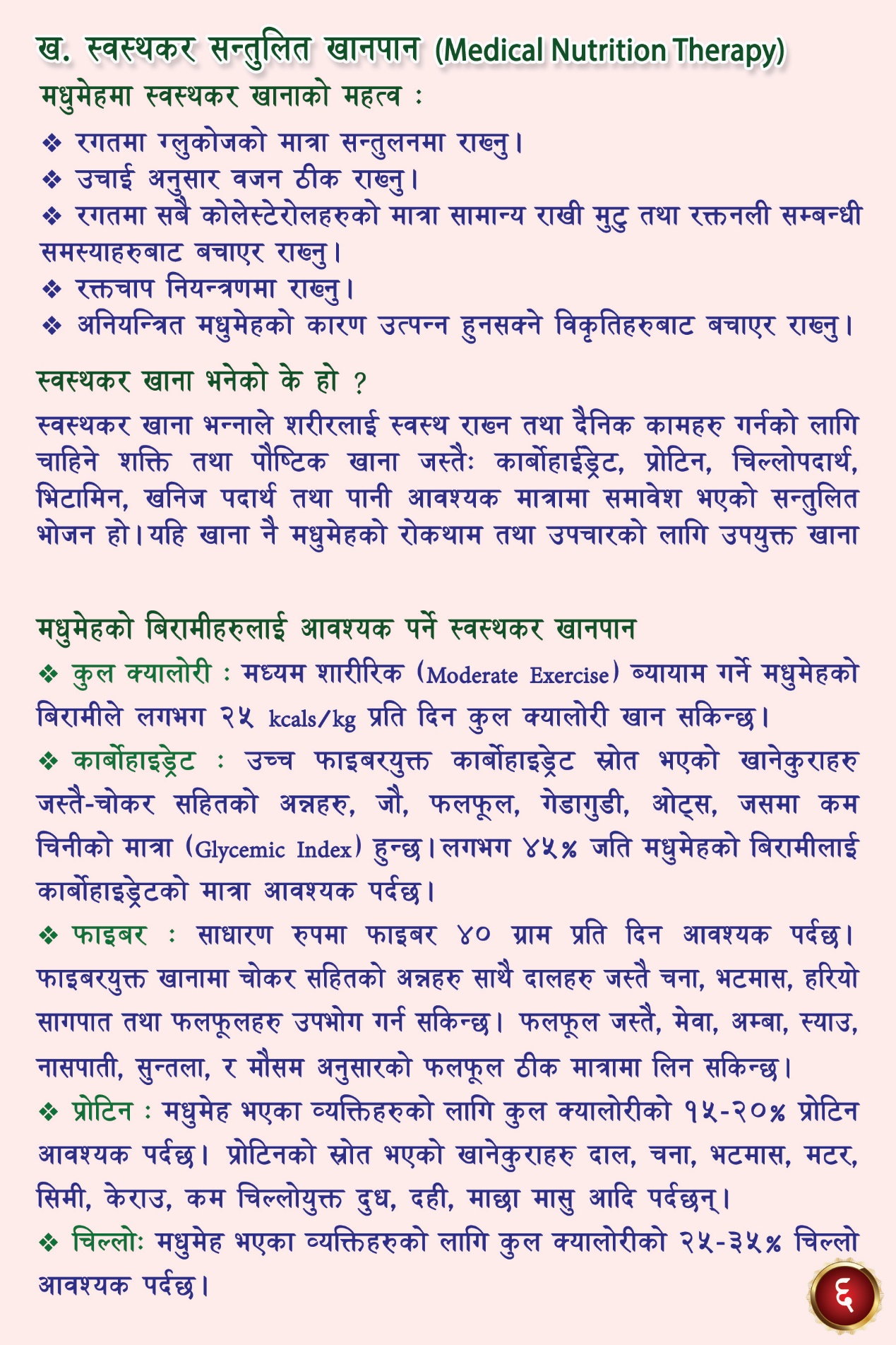


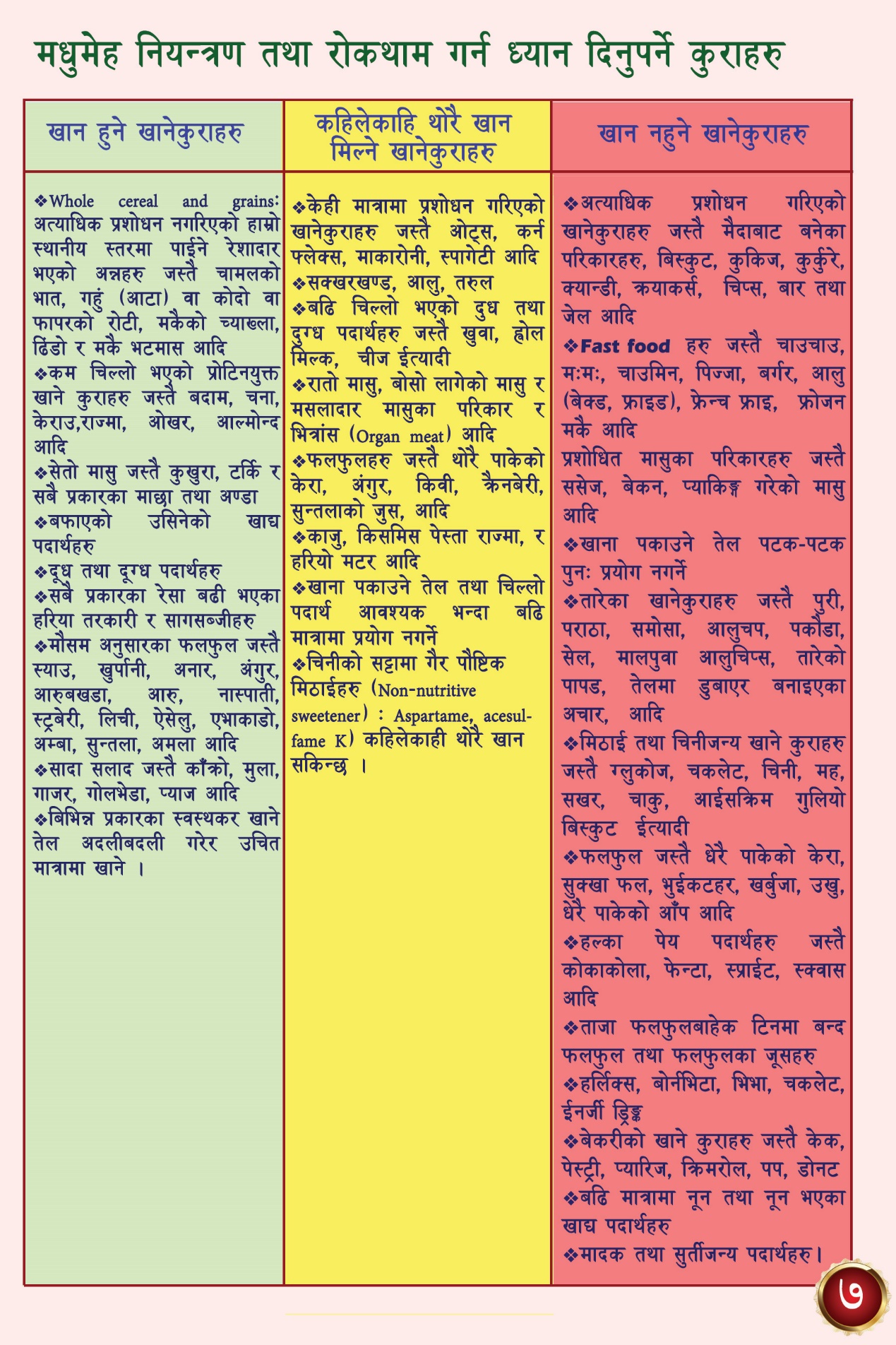


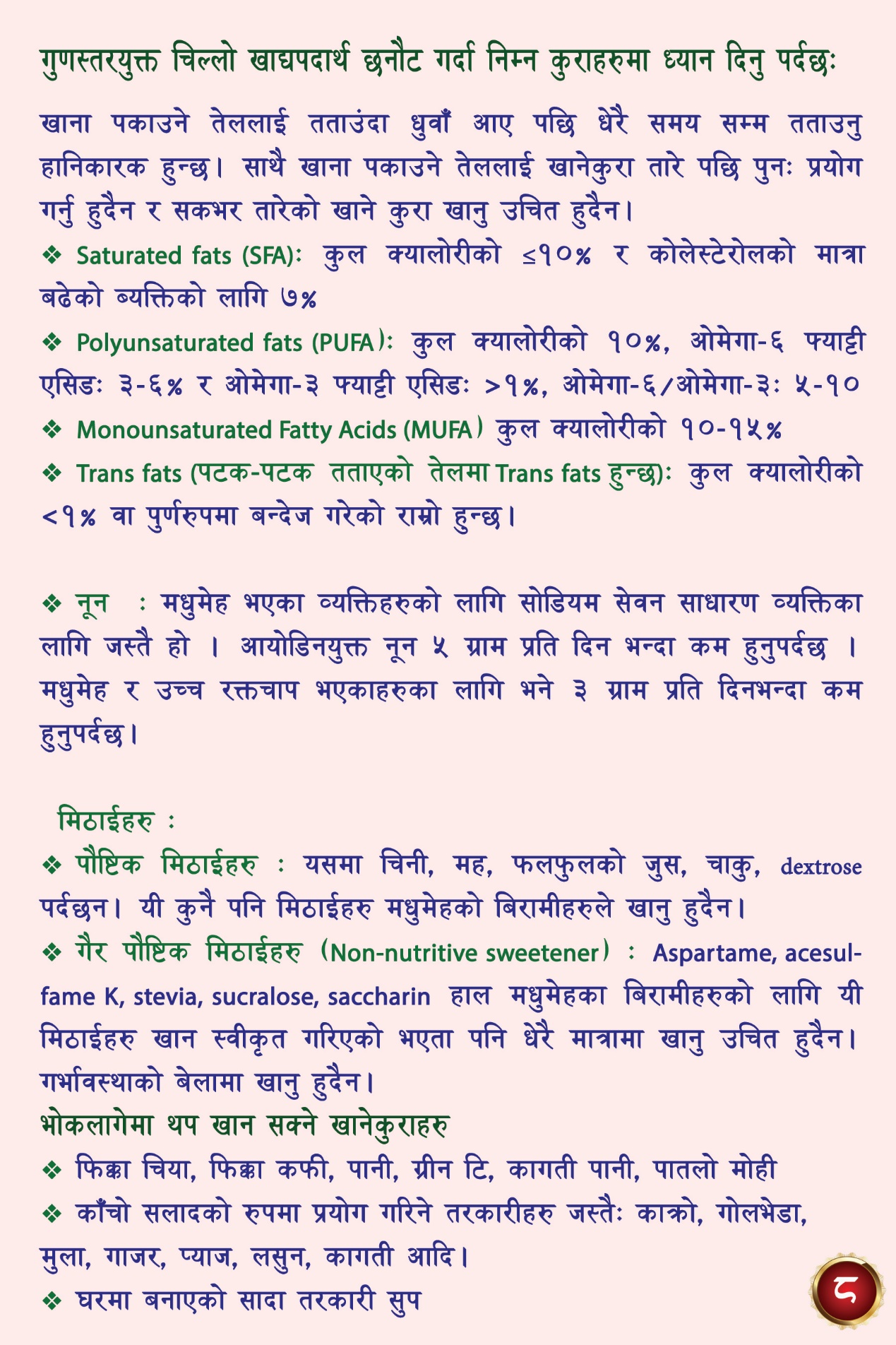


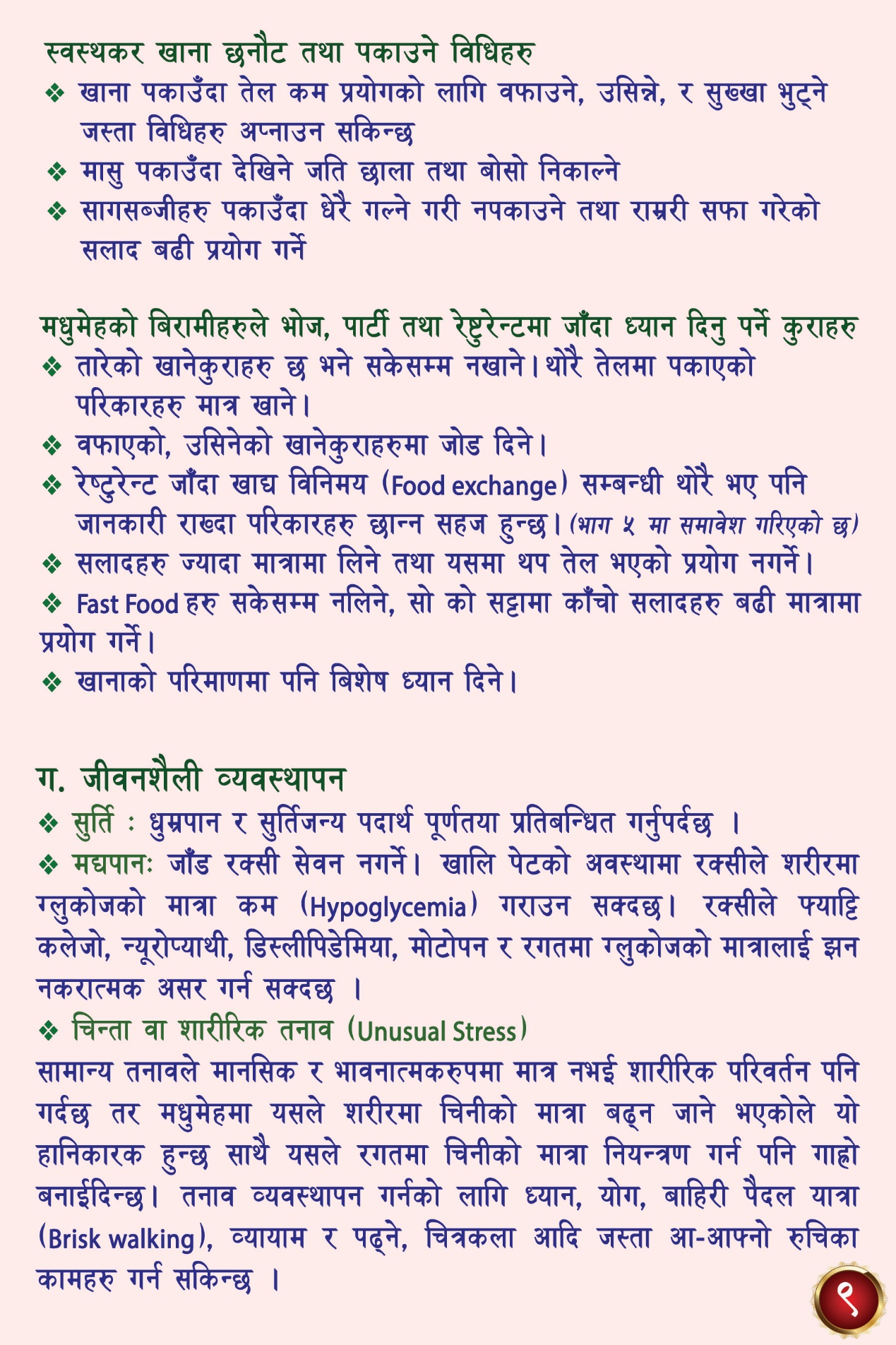


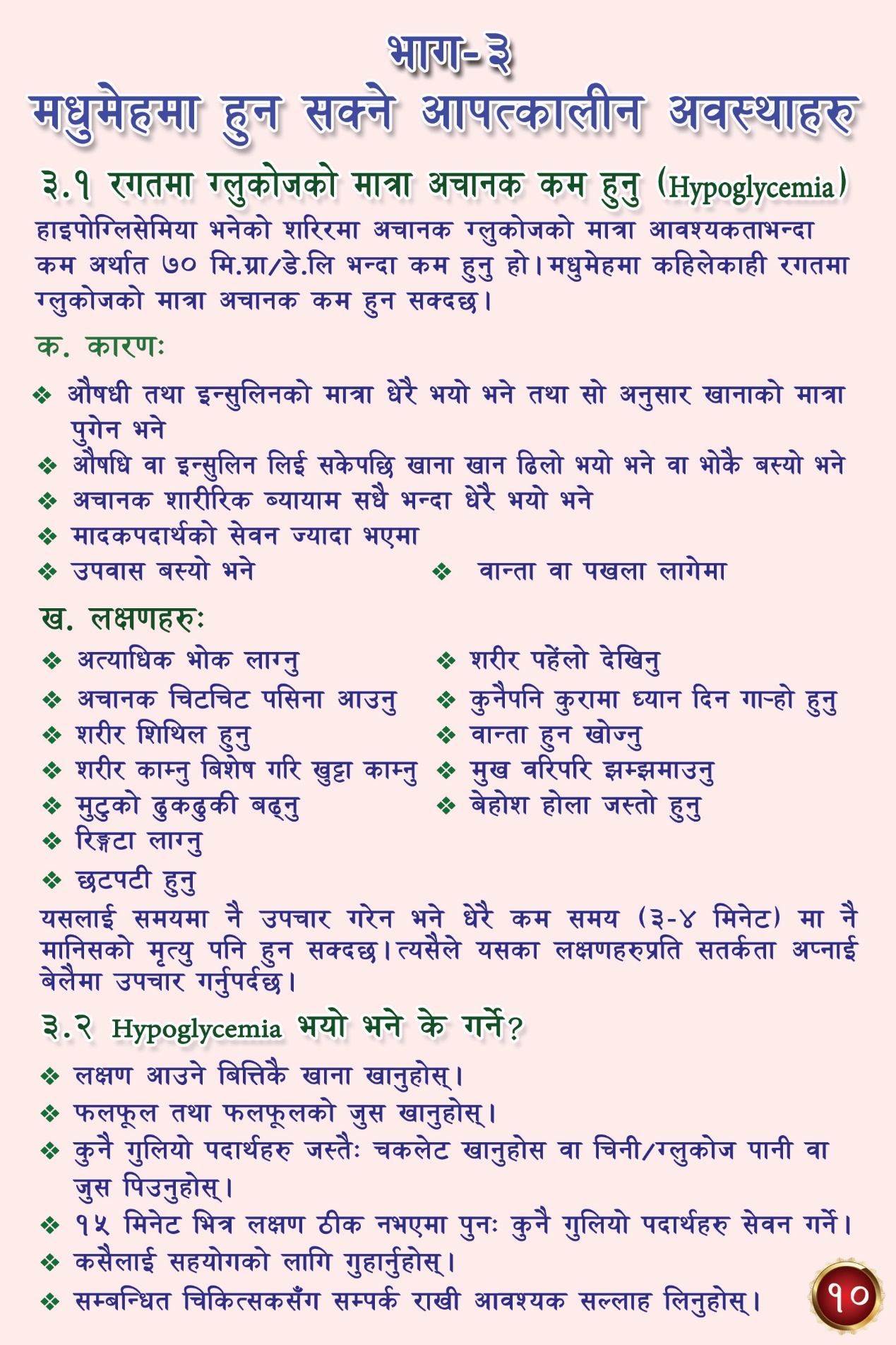


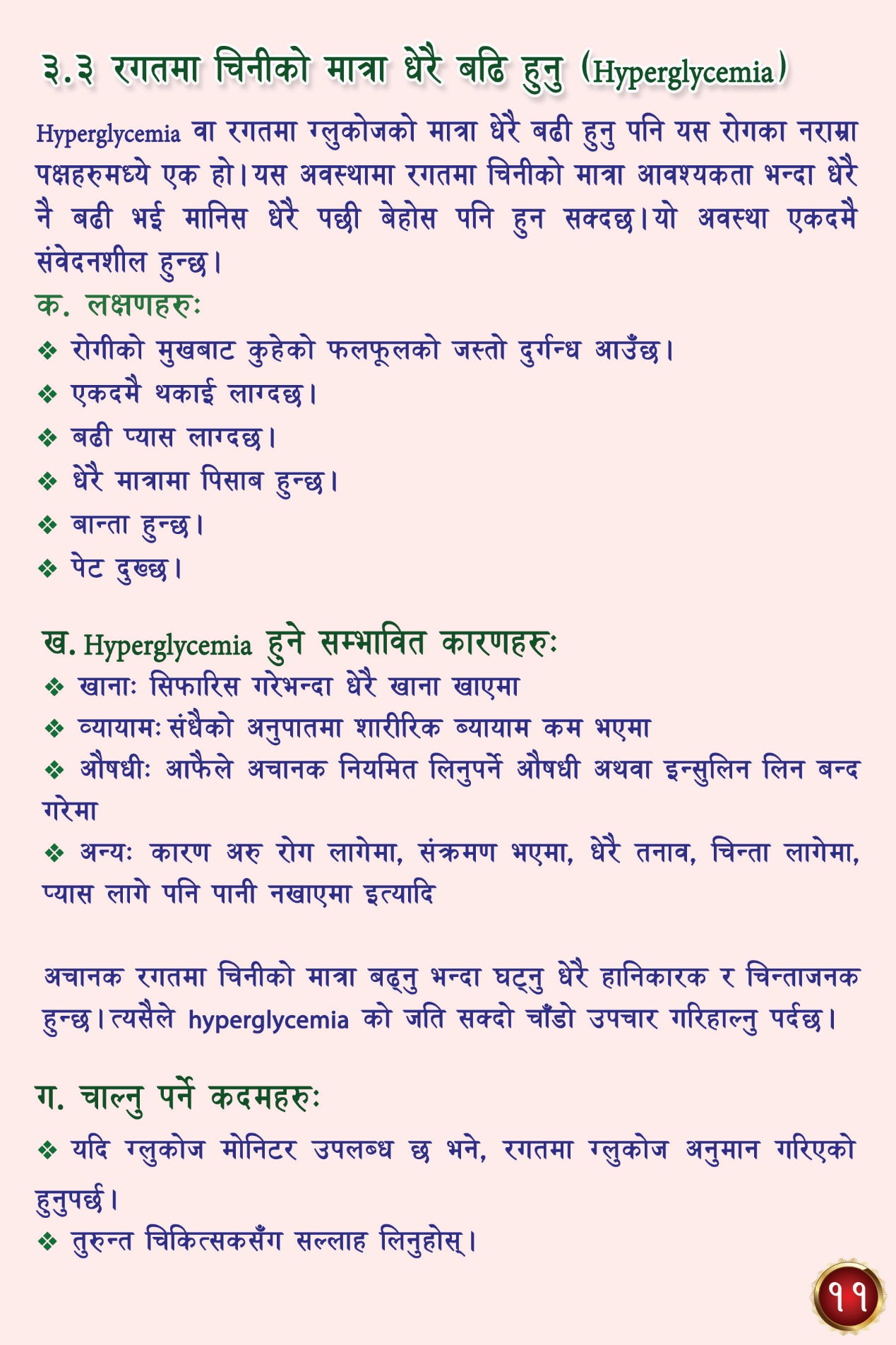


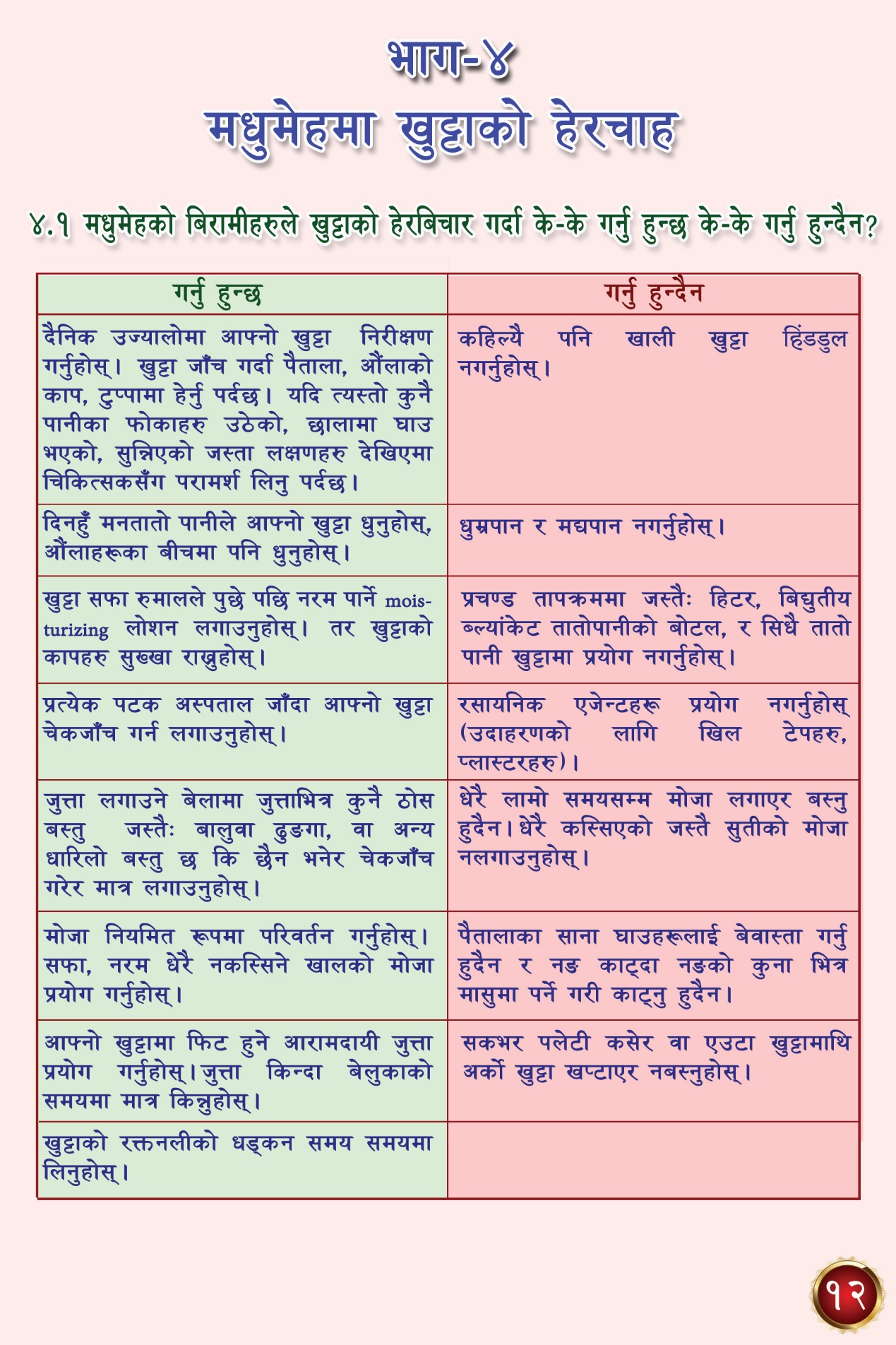


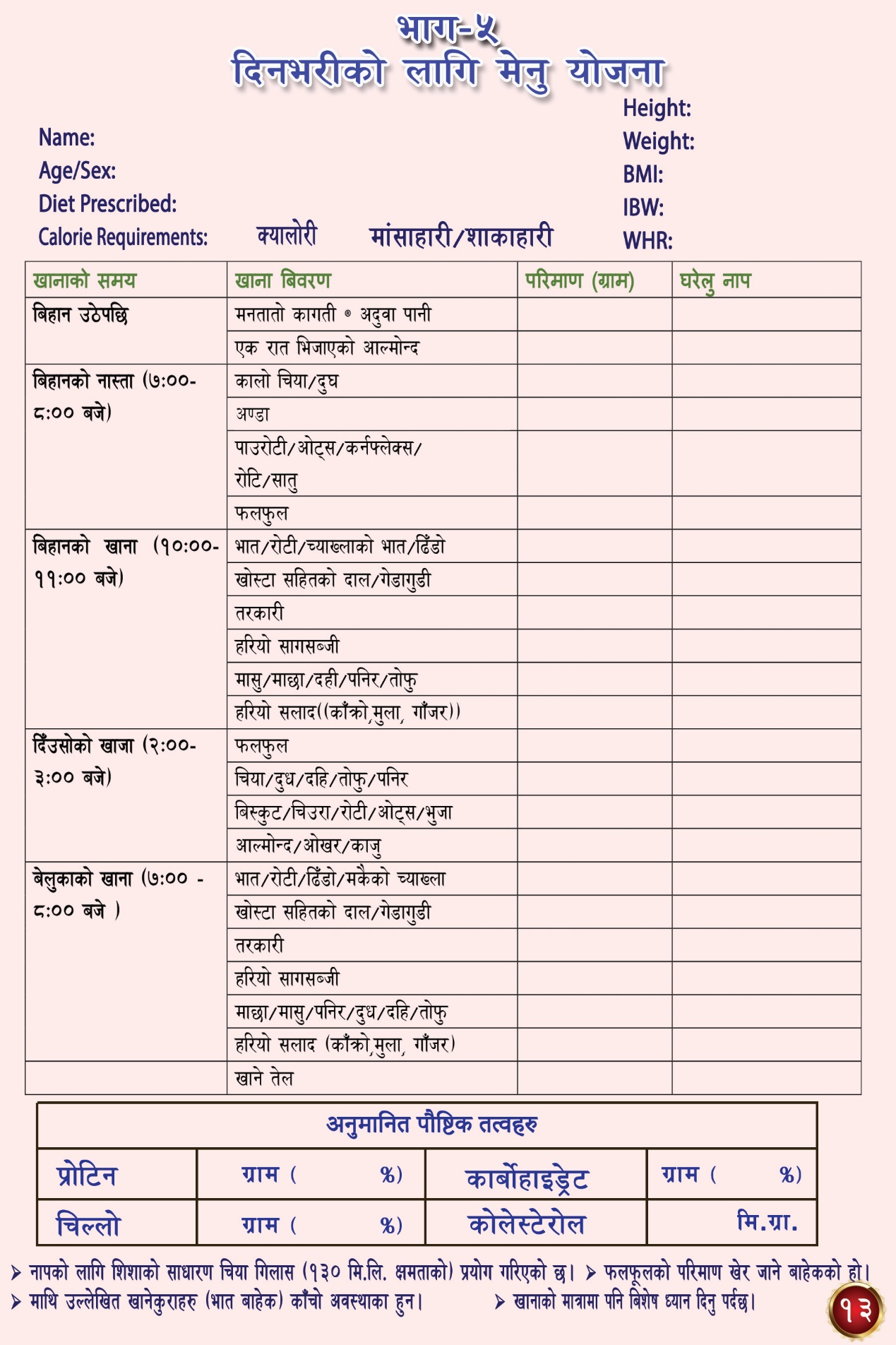


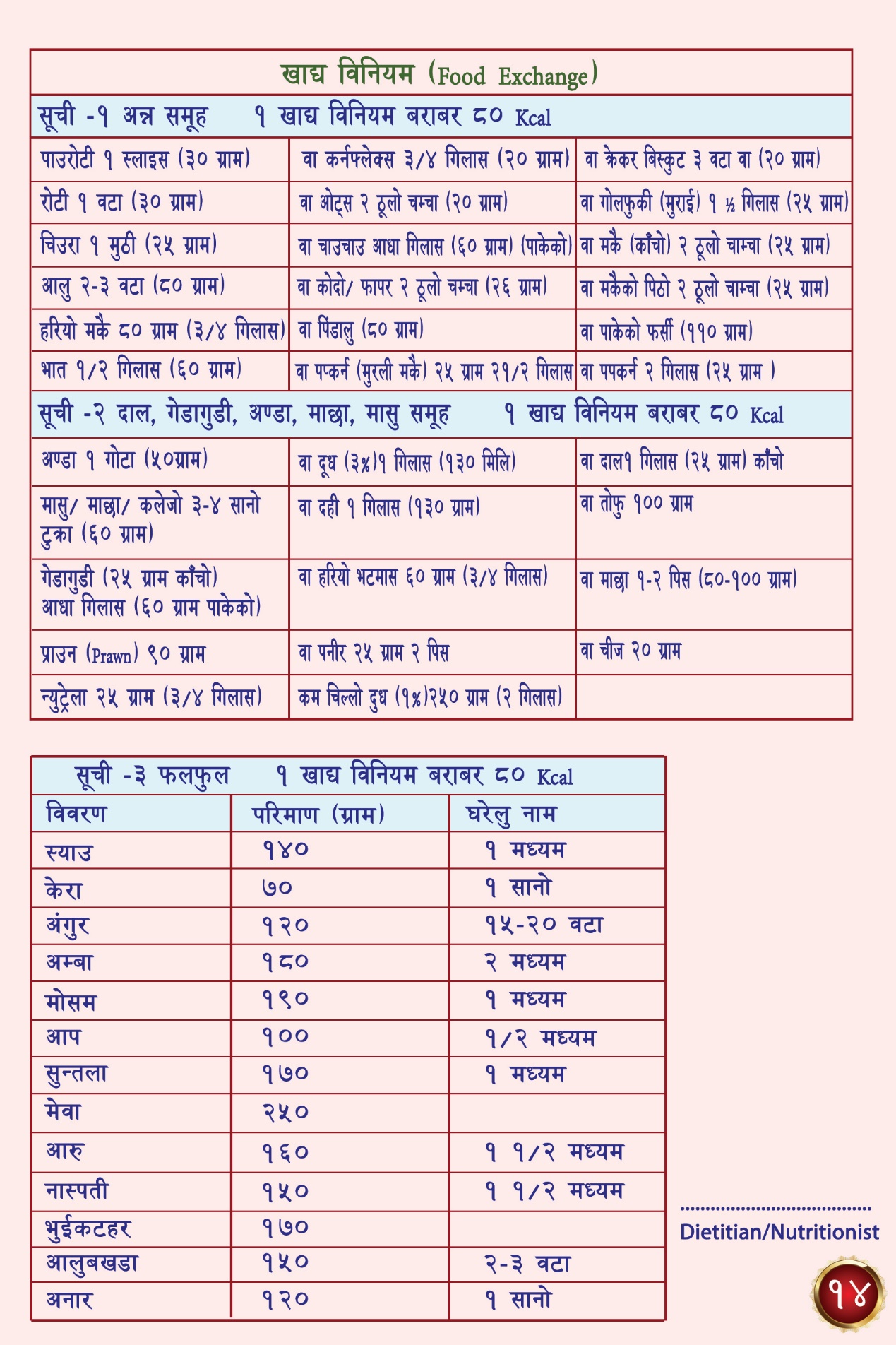


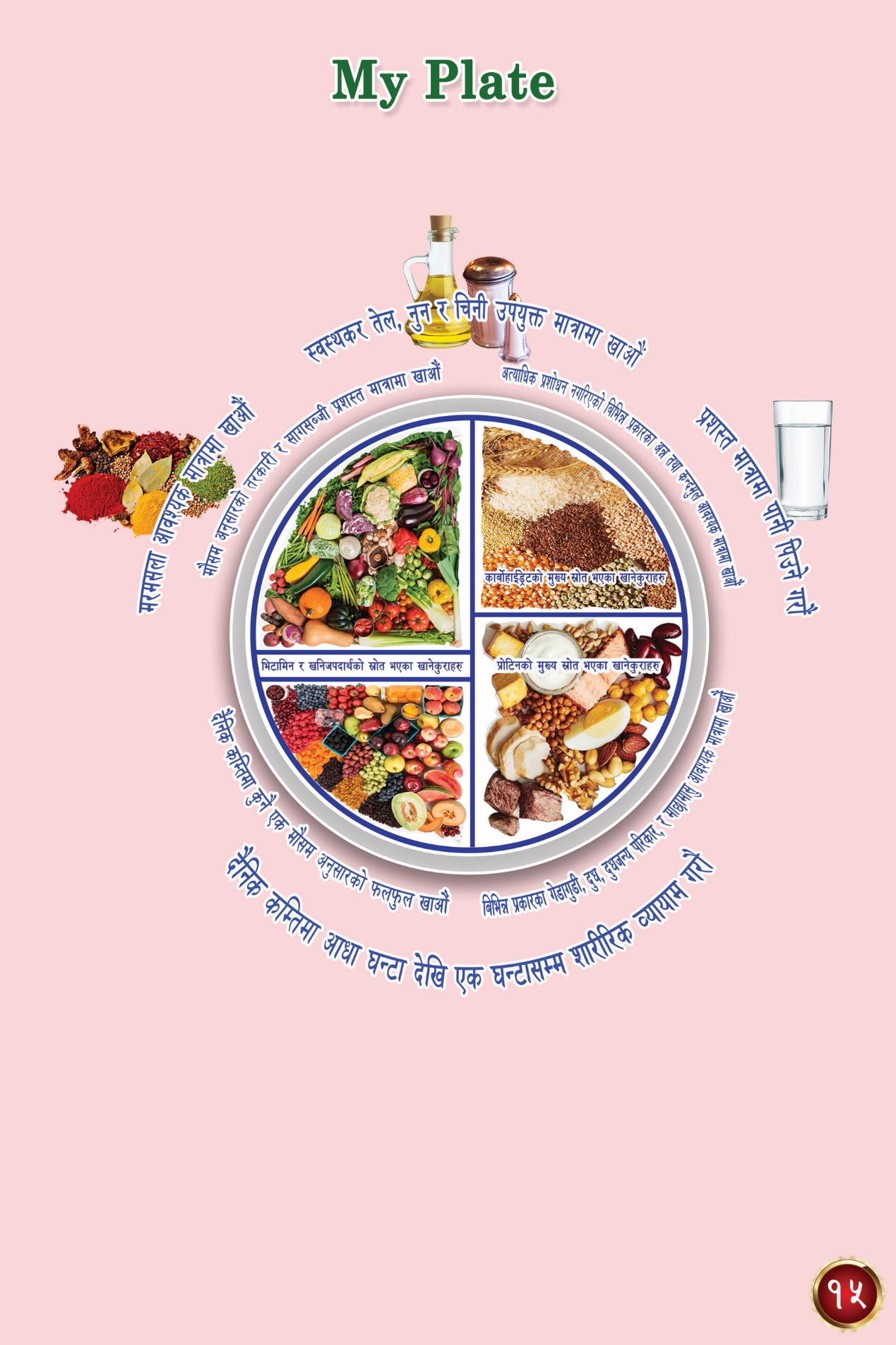


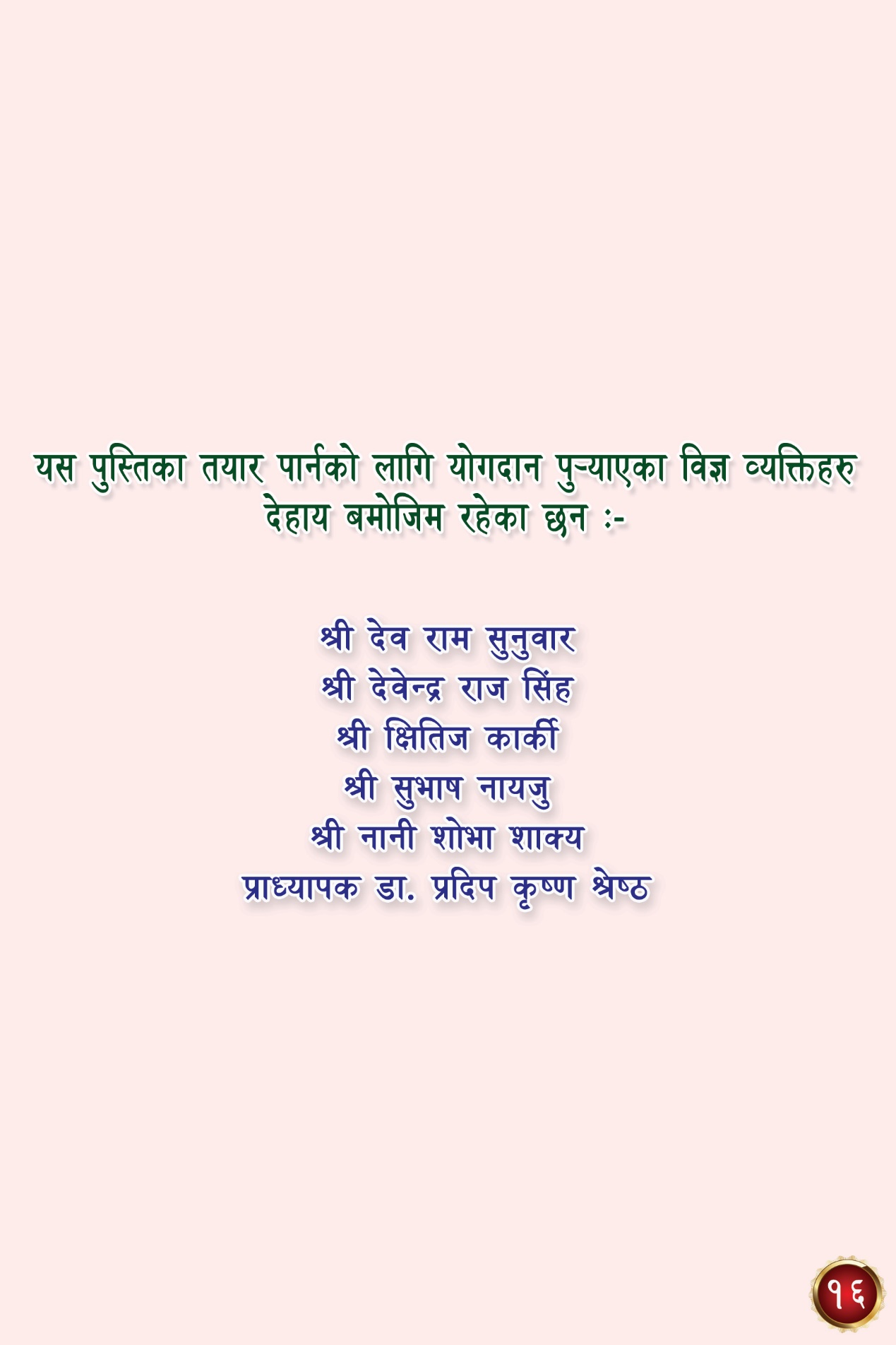


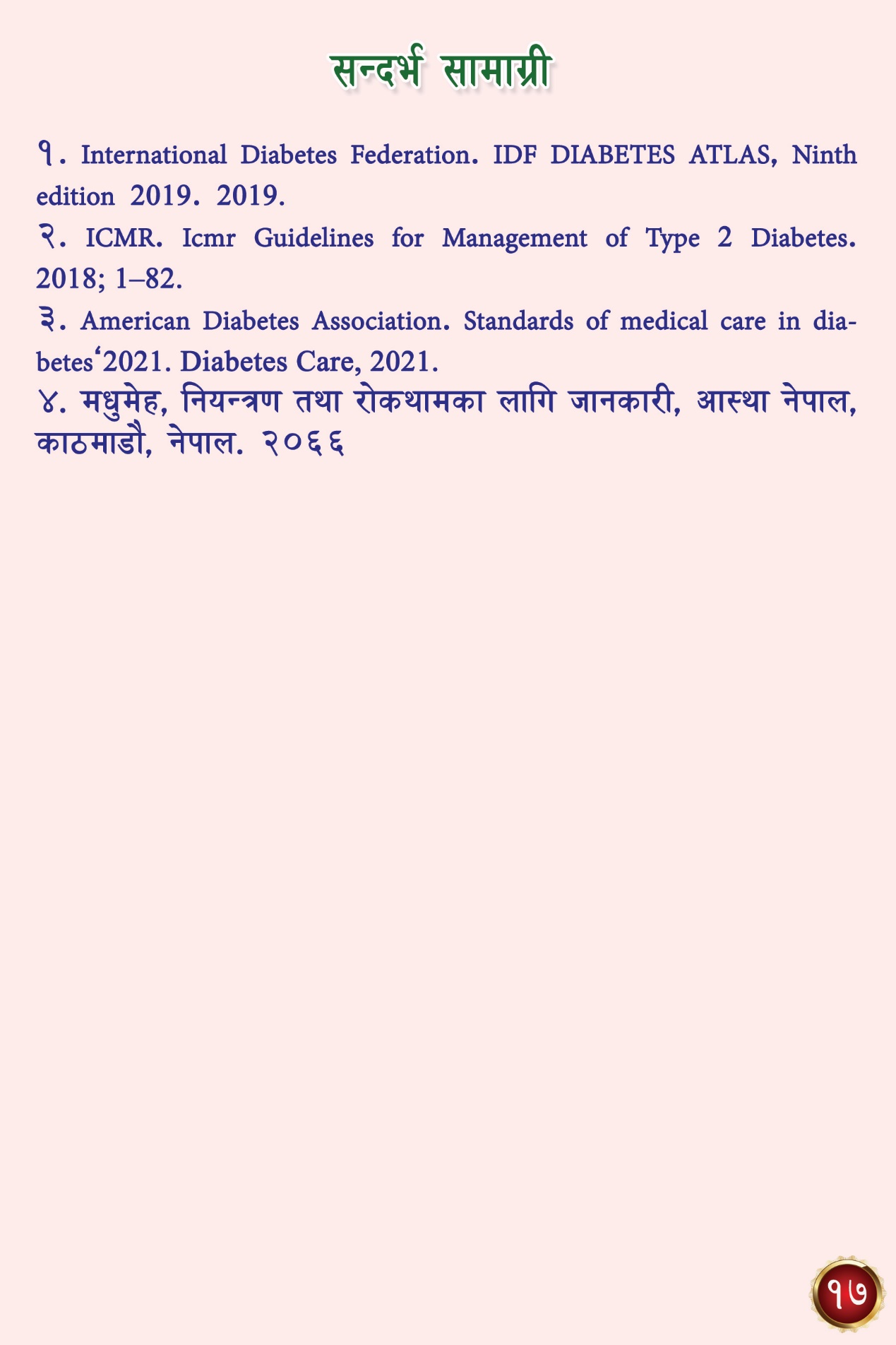

Supplement: Supplementary File [file mmc1.docx]
