# Supplementary material for: Effectiveness of a dietician-led intervention in reducing glycated haemoglobin among people with type 2 diabetes in Nepal: a single centre, open-label, randomised controlled trial
Source: Lancet Reg Health Southeast Asia. 2023 Sep 25;18:100285. doi: 10.1016/j.lansea.2023.100285 (PMC10667281; doi:10.1016/j.lansea.2023.100285)
Supplement: Study Protocol [file mmc2.docx]

**Effectiveness of a dietician-led dietary intervention in reducing Glycated Hemoglobin among type-2 diabetes patients: an open-label, randomized controlled trial**

**RESEARCH PROPOSAL**

*Submitted to*

**Institutional Review Committee (IRC)**

Institute of Medicine

Tribhuvan University

Maharajgunj, Kathmandu, Nepal

Email : [irc@iom.edu.np](mailto:irc@iom.edu.np)

Telephone : 01-4413187

**For approval of research proposal and ethical clearance**

*Submitted by*

Name : Mr. Devendra Raj Singh

Full Qualification : MSc. HP&PH, MA

Designation : Associate Professor

Department : Public Health

College/Org. : Asian College for Advance Studies, Purbanchal University

Address : Satdobato, Lalitpur

Contact no. : 9851366558

Email : [dsingh3797@gmail.com](mailto:dsingh3797@gmail.com)

**Detail information of investigators**

**Principal Investigator**

| **S.N.** | **Full name** | **Details** | **Signature** |
| --- | --- | --- | --- |
| 1. | Mr. Devendra Raj Singh | Designation : Associate Professor  Full qualification : MSc. HP&PH, MA  Department : Public Health  Campus/Institute/Org. : Asian College for Advance Studies, Purbanchal University  Mobile no. : 9851366558  Email : [dsingh3797@gmail.com](mailto:dsingh3797@gmail.com) |  |

**Co-investigators**

*(Please also indicate as Guides and Co-guides under the full names for thesis proposal)*

| **S.N.** | **Full name** | **Details** | **Signature** |
| --- | --- | --- | --- |
| 1. | Mr. Dev Ram Sunuwar | Designation : Assistant Professor  Full qualification : M.Sc. Nutrition and Dietetics  Department : Public Health  Campus/Institute/Org. : Asian College for Advance Studies, Purbanchal University  Mobile no. : 9851147339  Email : [devramsunuwar@gmail.com](mailto:devramsunuwar@gmail.com) |  |
| 2. | Dr. Matina Sayami | Designation : Lecturer  Full qualification : M.D Internal Medicine  Department : Endocrinology Unit, Department of Internal Medicine  Campus/Institute/Org. : Maharajgunj Medical Campus, Institute of Medicine  Tribhuvan University Teaching Hospital  Kathmandu, Nepal  Mobile no. : 9851091266  Email : [matinasayami@gmail.com](mailto:matinasayami@gmail.com) |  |

*(Please add rows if needed)*

**General information**

- Name and title of Supervisor (For graduate and post graduate students)
- Names and titles of Guide and Co-guides (For thesis)
- Address and telephone number(s) of the research site(s)
- Name(s) and address(es) of the clinical laboratory(ies) and other medical and/or technical department(s) and/or institution(s) involved in the research
- Name and address of the sponsor/funder if any.

# **Research title**

**Effectiveness of a dietician-led dietary intervention in reducing Glycated Hemoglobin among type-2 diabetes patients: an open-label, randomized controlled trial**

# **Project summary (~250 words)**

Diabetes mellitus is one of the major intractable public health problems in developing countries including Nepal. The dietary approach is of paramount significance in the management of type 2 diabetes. There is still a lack of evidence on a dietitian-led dietary approach to the management of T2DM patients in the Nepalese context. The aims of this study is to examine the effectiveness of dietitian-led dietary approach to management of diabetes on reduction of HbA1c level, nutrition education score and macronutrient intake among T2DM patients in a tertiary care hospital in Kathmandu, Nepal.

The Dietary Approach to the Management of Diabetes (DIAM-D) trial is a hospital-based, open-label, two-armed, randomized control trial. A total of 156 participants with T2DM having HbA1c >6.5% will be enrolled in the study. Participants will be consecutively enrolled and assigned to receive nutrition education and counseling (NEC) and diet plan in the intervention group (n=78) and usual routine care in the control group (n=78) randomly. The NEC will be provided to participants on group session and the diet plan will be face-to-face individual basis at the time of enrollment and follow-ups will be done every month. Baseline data will be collected using a structured questionnaire for an interview and the biochemical tests will be measured. Baseline data will be collected at the time of enrollment, midline in three months and end-line data collection in 6 months. The primary outcome of the study will be a difference in mean change (from baseline, midline to 6 months) in the HbA1c level between the two study arms from baseline to end line. The secondary outcomes measure will be changed in biochemical and clinical parameters between the two arms at baseline midline to six months. Data will be entered using Epidata Software and transferred to the STATA/MP version 14.1 for further analysis. Data will be analyzed using an intention-to-treat basis. Independent sampele t-test will be used to estimates changes between the intervention and usual care arms.

**Key word:** Dietitian, Dietary approach, Management, Diabetes, Nepal

# **Introduction**

Diabetes mellitus (DM) refers to the metabolic syndrome characterized by high blood glucose levels considering major public health problems globally ^1^. The global prevalence of diabetes in adults has been increasing over the decades ^2^. According to the world health organization (WHO), diabetes is the seventh leading cause of death accounting for 1.59 million deaths in 2016 ^3^. According to the International Diabetes Federation (IDF) data, it was estimated that among 18-99 years, there are 451 million people with diabetes globally. The number projected to increase to 693 million by 2045 ^4^. The rapid change of diabetes prevalence in many countries specially developing countries have been influenced by rapid urbanization and drastically changes towards the sedentary lifestyle^2^. Similarly, an increase in the prevalence of diabetes is characterized by various modifiable and non-modifiable risk factors, modifiable risk factors include changes in dietary habits and sedentary lifestyle which contributed to overweight/ obesity and non-modifiable risk factors including genetic cause and age ^5^.

The prevalence of diabetes mellitus in the south Asian region was a rapid increase over the last two decades ^6^. According to the International Diabetes Federation (IDF) data for Nepal, the prevalence of T2DM in the 20-79 years age group was 4% in 2017 and the predicted number of undiagnosed cases was 532,100. IDF estimates the prevalence projected to increase by 6.1% and 1,264,200 undiagnosed cases in 2045 in Nepal^7^. Evidence from systematic review and meta-analysis done by Gyawali et al, (2014) also reported that the prevalence of diabetes is a major public health problem with a prevalence rate of 8.4% ^8^. Dhimal et al. conducted a nationwide cross-sectional population-based study carried out from 2016-1018 found that the prevalence of diabetes was 8.5%^9^. Diabetes is the third most common non-communicable disease in Nepal, which causes 12% of all hospitalizations^10^.

# **Rationale and Justification of study**

The prevalence of type 2 diabetes mellitus (T2DM) has drastically increased in recent years in Nepal^11^. T2DM is accompanied by complications such as dyslipidemia, hypertension, and obesity. Several studies have shown that these complications can be prevented if lowering the HbA1c level and which has to be maintained within normal limits^12^.

Dietary management is considered as the cornerstone of blood glucose level control in T2DM patients^13^. Nowadays, nutrition education and counseling are the widely accepted strategies for the management of diabetes^14^. Various studies have shown that T2DM patients are an association with nutrition education and improving dietary habits, nutritional knowledge and improving clinical outcomes such as lower blood glucose and HbA1c levels^13–15^. Once the diagnosis is confirmed to T2DM, patients are managed by mainly three approaches such as diet alone, diet and drugs or diet and insulin^16^. Indeed, dietary factors are crucial for the management and prevention of T2DM ^13^. Furthermore, individualized nutrition education and counseling are effective strategies in adherence to diet recommendations and in improving glycemic control^12^. Dietitian-led nutrition education and lifestyle intervention are effective in reducing body weight and glucose-related outcomes^1^. However, limited studies have been carried out to examine the effectiveness of a dietary approach to the management of diabetes consulted by a dietitian in developing countries ^17,18^. Therefore, there is still a lack of evidence on effective guidelines and protocols for a dietitian-led dietary approach to the prevention and management of T2DM patients.

# **Objectives**

## **General objectives:**

The main objective of this study is to evaluate the effectiveness of dietitian-led dietary approach to management of diabetes on reduction of HbA1c level, nutrition education score and macronutrient intake among T2DM patients in a tertiary care hospital in Kathmandu, Nepal.

## **Specific objectives:**

- To measure the change in HbA1c level among T2DM patients between the intervention and control/usual care arm.
- To evaluate the change in macronutrient intake level among T2DM patients in the intervention and usual care arm.
- To assess dietitian-led nutrition education and counseling on nutritional knowledge of diabetes among T2DM patients.
- To evaluate the change in biochemical parameters among T2DM patients in the intervention and usual care arm.
- To assess the nutritional status such as BMI and WHR among T2DM patients

# **Research questions/hypothesis**

Dietitian-led dietary approach to the management of diabetes will improve on HbA1c level, nutrition knowledge score and macronutrient intake level compared to usual care among type 2 diabetes mellitus patients in tertiary care hospital in Nepal.

# **Research design and Methodology**

This study will adhere to the Consolidated Standards of Reporting Trials (CONSORT) 2010 updated guidelines for reporting for RCTs ^19^.

## **7.1. Research method**

a. Quantitative

## **7.2. Types of study**

a. Experimental/Interventional: The proposed study is a randomized, controlled, open-label trial to evaluate the effectiveness of dietitian-led dietary approach to management of diabetes on reduction of HbA1c level and macronutrient intake among T2DM patients in tertiary care hospital in Kathmandu, Nepal (Figure 2). This study utilized two-arm RCT with equal allocation of participants between the intervention and control group/usual care arm. The intervention will include a dietitian-led nutrition education, counseling session and individual basis diet plan emphasis on glycemic control diet targeted to T2DM patients attending during the OPD visits in hospital.

## **7.3. Study population/Sampling frame**

The source of the population of the study in both intervention and control group will be known cases of T2DM and newly diagnosed cases of T2DM patients who are within 25-64 years of age group attending the OPD of the hospital.

## **7.4. Study site and its justification**

The study will be conducted in Tribhuvan University Teaching Hospital (TUTH) in Kathmandu, Nepal where superspecialist services is available with high patients flow.

## **. Sampling method**

1. **Non probability:** This study will utilize non-probability purposive sampling methods to choose the tertiary care hospital in Kathmandu metropolitan city.
2. **Probability sampling:** Simple random sampling will be used to select the desired number of participants.

## **. Sample size**

Since, there was no similar research carried out in Nepal thus the sample size has been calculated based on the similar study conducted by JW Muchiri et al., (2010) in South Africa where, mean+ SE of HbA1c percentage in both intervention and control group was 9.67±0.29 Vs 10.30±0.29 respectively ^20^. Based on this consideration, JW Muchiri et al., (2010) data will be used to calculate the sample size taking α level of significance at 5% and power of 90%. The sample size will be calculated using test comparing independent two means in STATA/MP version 14.1 (StataCorp LP, College Station, Texas). The following formula with 5% significance level (Zα/2 = 1.96) and 90% power (Zβ) will be used.

Where

Z_α_ = Z value for α error

Z_β_ = Z value for β error

S = Common standard deviation between two groups

d = clinically meaningful difference

Z_α_=1.96, Z_β_= 1.282, S= 0.1, d= -0.64

The sample size will be calculated 108 (54 participants in each arm). Thus, the sample size will be adjusted with 20% non-response rate and 25% loss through follow-up and drop out the total sample size will be 156 (78 participants in Intervention and 78 participants in usual care arm respectively.)

## **7.7. Inclusion and Exclusion criteria**

**Inclusion Criteria:**

- Diagnosis of type 2 diabetes
- HbA1c>48 mmol/mol (6.5%) at time of diagnosis ^21^
- male or female aged ≥24-64 years and visited in OPD of target hospital
- Those who give consent to participate in our intervention study

**Exclusion Criteria:**

- Those who are pregnant women, lactating women or planning to become pregnant during

the course of study.

- Those who are severely ill or more than two comorbidities
- Those who have any plan to migrate from the study area for at least 1 year
- HbA1c ≥93mmol/mol (10.5%)
- Those who are on insulin therapy

## **7.8. Study variables**

**Dependent variable:** HbA1c level

**Independent variables**

- - **Socio-demographic information (from Baseline):** Age, Sex, Education level, Occupation, Religion, Marital status, Residence, Smoking habit, and Alcohol habit
  - **Clinical and biochemical parameters (baseline end line):** HbA1c, fasting blood glucose, Systolic diastolic blood pressure, Lipid profile, Quality of life, Body mass index (BMI), Waist hip ratio (WHR), Physical activity level, Current medication used
  - **Dietary intake (baseline end line):** Carbohydrate, Fat, Protein, Food groups, Nutritional knowledge score on diabetes.

## **7.9. Expected time and duration of the study**

The study will be carried out for six months, from baseline data collection, follow-up midline data collection and end-line data collection for outcome measure.

## **7.10. Tools and techniques for data collection**

The baseline and end-line data collection will be performed in both intervention and usual care arm. A pre-tetsed culturally accepted and adapted, Nepali translated version tools and techniques will be used to conduct the study. A total of three data collection team who have graduated in health or nutrition science will be hired from the local area.

**Tools and procedure**

- Screening checklist: This checklist entails information regarding inclusion and exclusion criteria.
- Baseline questionnaire: The study participants’ information on Socio-demographic, such as age, sex, occupation, educational level, residence, religion, ethnicity, etc. will be taken by using a face-to-face structured questionnaire interview. The questionnaire will be adopted from NDHS 2017 and STEP Survey 2019^22,23^.
- Dietary intake: Dietary intake assessment will be assessed before intervention (baseline) and after intervention (end line) using 24-hour qualitative dietary recall and food frequency questionnaire (FFQ) to compute the macronutrient intake level.
- Biochemical measurements: A fasting blood sample for HbA1c, fasting glucose, liver function test (bilirubin, ALT, AST ALP, and albumin) and lipid profile (total cholesterol, triglycerides, HDL, LDL) will be measured at baselines, three months and six months. All blood samples will be taken and handled, analyzed and disposed of according to local practice policy.
- Quality of life: The Problem Areas in Diabetes (PAID) score^24^, a 20 items questionnaire measuring the problems related to emotions, treatment, food and social support, will be measured at baselines and 12 months.
- Physical measurement: Height will be measured using a stadiometer to the nearest 1cm. Participants' weight will be measured to the nearest 0.1kg using the Seca weighing scale. BMI will be measured calculated using the widely used formula (BMI= weight (kg)/height (m)^2^), and waist Hip Circumference (WHR) will be measured using measuring tape. Systolic and diastolic blood pressure will be measured three times after 5 minute seated rest using a sphygmomanometer and stethoscope. The trained data enumerator will be taking physical measurements according to the study manual procedures.
- Nutritional knowledge score on diabetes: The diabetes knowledge test (DKT)^25^will be used to assess the nutritional knowledge score on diabetes.
- Physical activity: The physical activity will be measured using the Global Physical activity questionnaire recommended by WHO STEPwise surveillance tools^23^.
- Monitoring checklist: This will be used as every month follow up checklist. The information on lifestyle modification such as consumption of food, physical activities, adherence, and diabetic-related minor and major complication will be sought.

## **7.11. Management protocol of patients/participants if applicable**

In the intervention phase, the educational and diet plan package will be developed. All the intervention packages will be approved by a multi-disciplinary expert team comprising an endocrinologist and dietitian prior to the intervention. Patients in the usual care arm will receive routine care as to how he/she is practicing in their daily life. Routine care includes general knowledge on diabetes disease process, blood glucose monitoring, a healthy lifestyle, preventing treating diabetes complications and developing personalized strategies for the decision-making process ^26^. The intervention package will include mainly two-phase, nutritional counseling emphasis on lifestyle modification of diabetes patients and individual basis diet plan for each T2DM patients for intervention arm. The nutrition education and counseling and individual diet plan will be applied by trained dietitians.

In the first phase, participants in the intervention arm will receive a nutrition counseling session. Nutrition counseling package consists of six modules (knowledge about diabetes and additional information, treatment of diabetes, food-based dietary guidelines, and exercise guidance, knowledge of hypoglycemia treatment, foot care, medication, and blood glucose monitoring)^12,16^ which will be developed as a training manual and approved by multi-disciplinary expert team. Nutritional counseling will be provided by a dietitian who had to work at the participating working center. Every month, follow-up will be done to reemphasize the key message.

In the second phase, all patients in the intervention arm will be provided an individualized diet plan to reinforce the concept of controlling the serving size of foods every month which will be lasted for 45-60 minutes. During the patient's visits to OPD, the dietitian will obtain daily nutrient intake by asking the patients to recall the foods and beverages consumed for the past 24 hours. Each participant will be given an individual basis diet plan within the limit of the acceptable macronutrient distribution range (AMDR) ^20^. Energy requirements will be prescribed by a dietitian for each participant based on the patient's height, weight, and physical activity level according to the guidelines of the American diabetic association (ADA)^26^. Energy distribution will be set in accordance with the limit of AMDR i.e. protein 10-35%, fat 20-35%, and carbohydrate 45-65%)^20^. This guideline will be developed for the Nepalese population based on the ADMR compositions includes the use of a food exchange portion in diabetic diet planning. One piece of food exchange portion is defined as one serving for every 80 kcal of a food product. Thus, a similar type of food exchange portion can be exchanged where the nutritional value is almost the same. The individual total energy required per day is calculated according to the individual weight and daily physical activity level using Harris-Benedict equations. Balanced diet for each patient will be planned and the total day's diet plan is distributed into five meal patterns such as breakfast, launch, snack, and dinner and a bedtime snack. In order to avoid excessive energy intake and assure a balanced diet, we emphasize moderate carbohydrates, moderate fat foods and consuming foods rich in fiber and micronutrients. Follow up will be done every month. All educational materials will be developed in English and then translated into Nepalese language.

Table 1 Content of Nutrition Education and Counseling Module for DIAM-D trial

| SN | Topic | Module |
| --- | --- | --- |
| 1 | Knowledge about diabetes mellitus | Basic concept of nature of diabetes, causes and risk factors, types, clinical features, insulin action, body response to food in diabetes and non-diabetes state and complication |
| 2 | Treatment of diabetes | Diet alone, diet and drugs or diet and insulin, exercise and their  role in treatment of diabetes |
| 3 | Food-based dietary guidelines, healthy eating pattern | Dietary guidelines for each food group such as starch, fruits and vegetables and additional information on diabetes.  A nutrition education brochure will be provided. Information will be emphasis on healthy eating habits such as information about glycemic index and glycemic load of food, the difference between simple and complex carbohydrate, difference between saturated  and unsaturated fat, total calorie intake according to their needs, and calorie contains in beverage and alcohol  Overview of food groups and their roles in the body. |
| 4 | Diet planning: serving size and meal frequency | Guidelines of serving size and meal frequency and its importance  Demonstration: serving size (household measurement) |
| 5 | Knowledge of hypoglycemia treatment  and foot care | Guidelines of knowledge of hypoglycemia and its treatment knowledge of foot care practices |

## **7.12. In case of RCT, process of randomization and blinding, description of stopping rules for individuals for part of the study or entire study, the procedures and conditions for breaking the codes etc.**

The dietitians and enumerators will explain the purpose of the study to the participants and those participants who willing to give written informed consent and meet the inclusion criteria will be randomly assigned to either the intervention or the usual care arm. Once participants sign an informed written consent form, he or she will be enrolled in the study. Eventually, participants will be enrolled in either intervention or control groups randomly using computer generated random numbers.

## **7.13. A graphic outline of the study design and procedures using a flow diagram including the timing of assessments.**

Figure 1. Study design


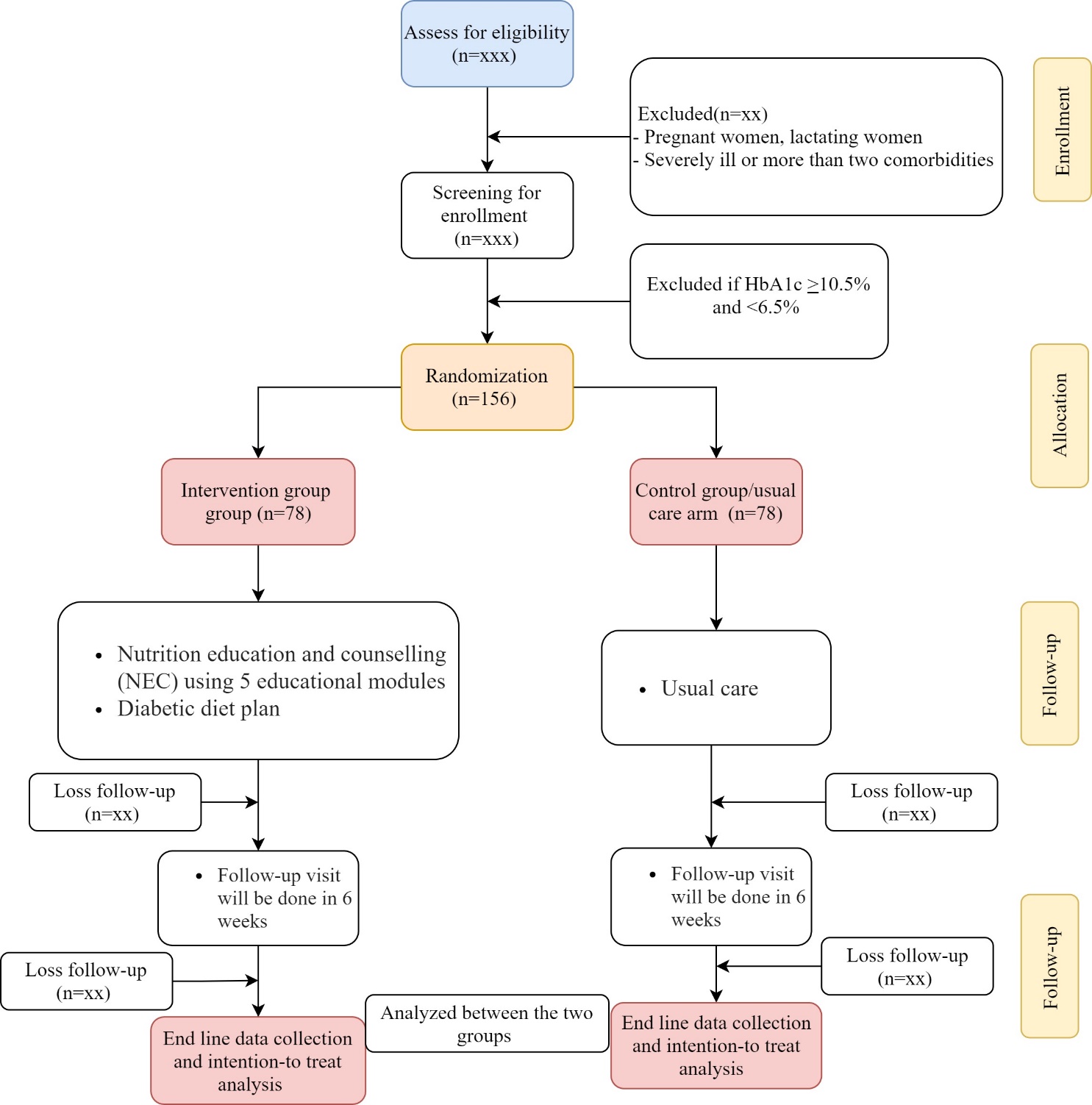


# **Plan for data management and statistical analysis**

- **Data entry:** A data entry program will be developed using EpiData version 3.0. A number of skips and ranges will be developed to ensure data entry. Trained data entry professionals will enter the data. After the data entry, a thorough check and cleaning of data will be done to maintain consistency.
- **Data analysis:** Data will be analyzed on an intention-to-treat analysis. Data will be analyzed using STATA/MP version 14.1 (StataCorp LP, College Station, Texas). A number of statistical analysis will be done to analyze the data which are listed below:
- **Normality test:**The normality of data will be assessed using the Kolmogorov-Smirnoff test and visual inspection of Q-Q plots. Normally distributed data will be presented as means with standard deviation whereas non-normally distributed data will be expressed as medians with interquartile ranges.
- **Descriptive statistics**: Descriptive statistics will be carried out for socio-demographic information. Frequency, percentage, mean (standard deviation) and median (inter-quartile range) will be calculated under descriptive statistics.
- **Chi-square test**: Chi-square or Fisher exact test will be used to analyze between the intervention and usual care arm for categorical variables.
- **Independent sample t-test:** Difference between intervention and usual arm will be applied using independent sample t-test assuming equal variance if data is normally distributed for continuous variables (two-tailed; parametric).
- **Mann-Whitney U test**: Mann-Whitney U test will be used to compare the medians of continuous variables if in case the assumption for independent sample t-test will be violated (two-tailed; non-parametric).
- **Linear regression**: A linear regression analysis will be used to model the primary and secondary outcome measures.

# **Biases**

Due to the educational experimental study, selection bias can occur during the selection of control and intervention group. To minimize the selection bias, the randomization procedure will be strongly followed. Similarly recall bias can occur, while asking the question. To minimize it, proper probing will be carried out. Likewise, interviewer bias can occur, to minimize it length of time taken to interview the average intervention and the average control will be noted.

# **Limitations of the study**

In this study, the participants will be enrolled from OPD basis, so, we cannot resolve the adherence of dietary advice to all participants. Similarly, there may be a recall bias to collect 24-hour dietary recall and may have social desirability bias for collecting behavioral related history. To minimize recall bias, proper probing will be carried out. Likewise, interviewer bias can occur, to minimize it the length of time taken to interview the average intervention and the average control will be noted. Selection bias can occur during the selection process. We will strongly follow randomization to the selection of intervention and usual care arms by a trained data enumerator so that this will minimize the confounding. Also, the cross-contamination may occur between intervention and control arms. We will suggest the participants from the intervention arm not to share the provided information with the others to prevent cross contamination.

# **Safety considerations**

Safety of research participants will be ensured by well maintaining confidentiality of the personal information given by the participants.

# **Plan for supervision and monitoring**

The study team will make the necessary efforts to improve the validity and reliability of the study. The random sample selection process at each stage of the sampling procedure will ensure the representativeness of the study participants. The quality of the study will be assured at all stages of study design, data collection, and analysis. The questionnaire will be translated into Nepali and pre-testing will be done in 10% of the study sample to see the flow and wording of the questionnaire. Following points will be considered to assure data quality:

**Design phase**

- A detailed search of the relevant literature of the previously published documents will be done. Qualified and well-experienced faculty members have been proposed and enumerators for this study will be the students of final year Bachelor in Public Health and they will be also trained for conducting the study.

**Data collation phase**

- Firstly, thesis writing students will be trained for data collection procedure, participants’ selection and randomization. The manual of data collection will be developed and distributed to the enumerators and study team members. Conduct a week-long training workshop to the data enumerators and dietitian regarding data collection techniques and diet counseling. The study questionnaire, anthropometric instruments, biochemical instruments will be pre-tested. Pre-testing should be conducted in order to maintain the accuracy and clarity of tools.
- Nutritional counseling and diet plan guidelines will be pre-tested in another hospital not included in the study area. Pre-tested guidelines will be approved by a multi-disciplinary expert team.
- After pre-testing all the ambiguous, misleading and wrongly interpreted questions will be omitted and the questionnaire will be revised in accordance with the findings of pre-testing. Instruments such as biochemical tools and clinical tools will be regularly calibrated before use.
- Supervision and monitoring during the data collection will be done by the investigators.
- Confidentiality while taking the interview will be maintained in order to avoid bias.
- The core study team will be made responsible to maintain the appropriateness of sampling and data collection techniques. Interviews will be conducted into the local language and the responses will be coded simultaneously on the questionnaire. The filled-in questionnaire will be checked and edited each day by the supervisor at the data collection sites.
- The data collection will be carried out in close supervision of the research team. In order to maintain the quality of the data collection, the investigators will make at least 10% accompaniments and 20% back checks every day. Any error in data collection will be recorded in the investigator logbook and the enumerators will be informed of the errors and suggested the improvement measures.
- All the filled-up questionnaires will be safely stored in a locker.

# **Expected outcome of the research**

The results of this study are expected to show that the dietitian-led dietary approach to the management of diabetes will improve on HbA1c level, nutrition knowledge score and macronutrient intake level compared to usual care among type 2 diabetes mellitus patient in tertiary care hospital in Nepal.

# **Plan for dissemination of research results**

The findings of this study will be disseminated to the national and international scientific community, stakeholders and policymakers. The final report submitted will be submitted to the University Grant Commission (UGC). The results of this study will be also dissimilated through the scientific workshops, national and international conferences and published scientific papers in peer-reviewed index journals.

# **Plan for utilization of the research finding**

The proposed study is expected to deploy two final year students who will be writing their thesis for the partial fulfillment of Bachelor in Public Health course at Asian College for Advance Studies, Purbanchal University. The students who are expected to involve in this study are currently studying Bachelor in Public Health course at Asian College for Advance Studies under Purbanchal University. These students are supposed to conduct individual thesis for the partial fulfillment of the Bachelor in Public Health Course. As a part of this study, one will be conducting research entitled “Effectiveness of IEC materials on nutrition education and counseling for diabetes patients in Tertiary care hospital in Kathmandu”. Similarly, another student will be conducting research entitled “Dietary adherence, physical activity and nutritional status of diabetes patients in Tertiary care hospital in Kathmandu”.

During this study process, they will have opportunity to enhance their research capabilities on study design, sampling strategy, data collection techniques, development of nutrition education and counselling module, data management and analysis, drafting the report and dissemination of the study results in national and international conferences and publish research article in peer-reviewed index journal.

Furthermore, this study shed light on the role of dietitians in the management of diabetes. These study findings are also expected to inform others who want to management of diabetes by nutrition education, counseling, and healthy diets.

# **Work plan**

| **Gantt chart** | | | | | | | | | | | | | | | |
| --- | --- | --- | --- | --- | --- | --- | --- | --- | --- | --- | --- | --- | --- | --- | --- |
| S.N | Activities | **2019 A.D** | | **2020 A.D.** | | | **2021 AD** | | | | | | | | |
|  |  | **Nov** | **Dec** |  | | | **Jan** | **Feb** | **March** | **April** | **May** | **June** | **July** | **Sept** | **Oct** |
| 1 | Drafting proposal | **×** |  |  |  |  |  |  |  |  |  |  |  |  |  |
| 2 | Proposal submitted to UGC and under review |  | **×** | **×** | **×** |  |  |  |  |  |  |  |  |  |  |
| 3 | Submission and approve proposal to NHRC |  |  |  | **×** | **×** |  |  |  |  |  |  |  |  |  |
| 4 | Tools development |  |  |  |  | **×** |  |  |  |  |  |  |  |  |  |
| 5 | Nutrition counselling and diet plan guidelines developed. |  |  |  |  |  | **×** |  |  |  |  |  |  |  |  |
| 6 | Orientation to stakeholders  And guidelines approval |  |  |  |  |  | **×** |  |  |  |  |  |  |  |  |
| 7 | Training of thesis writing students |  |  |  |  |  | **×** |  |  |  |  |  |  |  |  |
| 8 | Sampling framework |  |  |  |  |  |  | **×** |  |  |  |  |  |  |  |
| 9 | Finalization of intervention and usual care arms in target hospital |  |  |  |  |  |  | **×** |  |  |  |  |  |  |  |
| 10 | Tools pre-testing and finalization |  |  |  |  |  |  | **×** |  |  |  |  |  |  |  |
| 11 | Finalization of research participants |  |  |  |  |  |  |  | **×** |  |  |  |  |  |  |
|  | Baseline data collection |  |  |  |  |  |  |  |  | **×** |  |  |  |  |  |
| 12 | Intervention |  |  |  |  |  |  |  |  |  | **×** | **×** | **×** |  |  |
| 13 | Midline data collection |  |  |  |  |  |  |  |  |  | **×** | **×** | **×** |  |  |
| 14 | Midline data analysis |  |  |  |  |  |  |  |  |  |  |  | **×** |  |  |
| 15 | Endline data collection |  |  |  |  |  |  |  |  |  |  |  | **×** |  |  |
| 16 | Endline data analysis |  |  |  |  |  |  |  |  |  |  |  | **×** |  |  |
| 17 | Literature review |  |  |  |  |  |  |  |  |  |  |  |  | **×** |  |
| 18 | Report writing |  |  |  |  |  |  |  |  |  |  |  |  |  | **×** |
| 19 | Dissemination of report |  |  |  |  |  |  |  |  |  |  |  |  |  | **×** |

# **Ethical issues and considerations regarding human participants**

The study will be conducted as per the guidelines formulated in the Declaration of Helsinki and ethical approval from the Nepal Health Research Council before commencing the research. This study will be registered with ClinicalTrials.gov. Every document used including questionnaire, consent, and information sheet will be submitted to the Board along with the methodology, study design, and evidence. After feedback, correction and modifications will be made into a questionnaire, and consent.

We will constitute a Data Safety Management Board (DSMB) comprising of an Endocrinologist/Physician, a dietitian, and one statistician. The DSMB members will prepare a study stopping rules and will review all the possible effects reported in the study on a quarterly basis. They will examine all participants' health status like comorbidities and decide if the study should be continued or terminated. After completed a follow up of 1/3rd of the study participants, DSMB will review data and recommend continuation, modification or termination. Any important protocol modifications, such as change of eligibility criteria, outcome analysis will be thoroughly communicated with members and will obtain amendments from NHRC.

# **Informed consent**

A written informed consent will be obtained from eligible participants. Voluntary informed participation and freedom of refusal at any time during study will be applied strongly. Participants can withdraw from the study anytime without giving reason and fear. Privacy and confidentiality of the collected information will be ensured at all levels. The purpose of the study, benefits, and harm to the participants will be properly explained in simple and understandable terms by the enumerator.

# **Budget**

| **Proposed Budget for the study** | | | | | | | **Justifications** |
| --- | --- | --- | --- | --- | --- | --- | --- |
| **S.N.** | **Description** | **Person** | **Months** | **LOE (%)** | **Rate NRs.** | **Total Amount (NRs.)** |  |
| **A** | **Personal Cost** |  |  |  |  |  |  |
| A1 | Principal Investigator | 1 | 14 | 0.3 | 2,500 | 10,500 | Since both principal investigator and Co-Investigators are full-time faculty at Asian College for Advance Studies, we have allocated minimum cost that includes minimum allowances to cover their necessary expenses. |
| A2 | Co-Investigator | 1 | 14 | 0.3 | 2,500 | 10,500 |  |
| A3 | Thesis writing students | 2 | 3 | 0.5 | 4,000 | 10,000 | This cost covers the minimum remuneration for thesis writing students who will involve in this study and work as an enumerators |
| A4 | Dietitian fee | 1 | 3 | 0.5 | 4,000 | 6,000 | Dietitian will be provided with a minimum fee for the patients counseling and diet plan to the study participants |
|  | **Sub-total** |  |  |  |  | **37,000** |  |
| **B** | **Laboratory cost** | **Person Items** | **Unit** |  | **Unit Cost** | **Total Amount** |  |
| B.1 | Bio-chemical test (testing service cost) | 156 | 2 |  | 600 | 202,800 | This cost covers the Biochemical tests charge during baseline, midline and end line. |
| B2 | Stadiometer | 2 | 1 | 1 |  |  | This equipment will be provided by Asian College For Advance Studies |
| B3 | Weighing Machine | 2 | 1 | 1 |  |  | This equipment will be provided by Asian College For Advance Studies |
| B4 | BP Set | 2 | 1 | 1 | 2,500 | 5,000 | This equipment will be provided by Asian College For Advance Studies |
|  | **Sub-total** |  |  |  |  | **207,800** |  |
| **C** | **Field Cost** | **Person** | **Days** | **Times** | **Unit Cost** | **Total Amount** |  |
| C1 | Travel cost | 2 | 72 | 1 | 100 | 14,400 | This cost is allocated for enumerator travel costs during the data collection period. |
| C2 | Daily allowances | 1 | 72 | 1 | 200 | 14,400 | Daily allowances for enumerators during the data collection period |
| C3 | Survey cost (tools pretesting) | 1 | 1 | 1 | 20,000 | 20,000 | Cost for tools pre-testing (Data collection tools, IEC materials, and menu plan sheet pre-testing ) |
|  | **Sub-total** |  |  |  |  | **48,000** |  |
| **D** | **Office cost** | **Event** | **Days** | **Times** | **Unit Cost** | **Total Amount** |  |
| D1 | Communication fee | 1 |  | 1 | 10,000 | 10,000 | This cost will cover the communication charge occurred during the study period |
|  | **Sub-total** |  |  |  |  | **10,000** |  |
| **E** | **Consultation services** | **Unit** | **Days** | **Times** | **Unit Cost** | **Total Amount** |  |
| F1 | Questionnaire, Nutrition Education Module, Diet plan sheet design, and sharing meeting | 1 | 1 | 1 | 20,000 | 20,000 | This cost is allocated for tools to develop, finalization meeting sharing workshop with study team and concern experts. |
| F2 | Workshop consultation on nutrition education module and diet plan among Dietitian | 1 | 4 | 1 | 5,000 | 20,000 | This cost is allocated for workshop consultation meeting for the approval of NEC and diet plan by multi-disciplinary expert team |
| F3 | Enumerators training/orientation | 6 | 1 | 1 | 1,000 | 6,000 |  |
| F4 | Data analysis | - | - | - | - | - | Analyzed by PI and CO-PI, therefore no cost is allocated. |
|  | **Sub-total** |  |  |  |  | **46,000** |  |
| **G** | **Students support** | **Person** | **Items** | **Times** | **Unit Cost** |  |  |
| G1 | Thesis Proposal preparation cost | 2 | 1 | 1 | 2,500 | 5,000 | This cost is provided to the students for their thesis proposal preparation |
| G2 | Thesis Preparation Cost | 2 | 1 | 1 | 2,500 | 5,000 | This cost is provided to the students for their thesis report preparation |
|  | **Sub-total** |  |  |  |  | **10,000** |  |
|  | **Total ( A+B+C+D+E+F+G)** |  |  |  |  | **358,800** |  |
| H | **Facilities and Administration Cost** |  |  |  |  |  |  |
|  | Overhead cost (10%) |  |  |  |  | 35,800 |  |
|  | **Sub-total** |  |  |  |  | **35,800** |  |
|  | **TOTAL STUDY COST without NHRC fee** |  |  |  |  | **394,680** |  |
|  | **NHRC (Ethical clearance) Fee 3%,** | **1** |  |  |  | **5,320** |  |
|  | **Grand Total** |  |  |  |  | **400,000** | **In words: Four lakhs only** |
|  |  |  |  |  |  |  |  |

# **References**

1 Sun Y, You W, Almeida F, *et al.* The effectiveness and cost of lifestyle intervention including nutrition education for diabetes prevention: A systematic review and meta-analysis. *J Acad Nutr Diet* 2018; **117**: 404–21.

2 Guariguata L, Whiting DR, Hambleton I, Beagley J, Linnenkamp U, Shaw JE. Global estimates of diabetes prevalence for 2013 and projections for 2035. *Diabetes Res Clin Pract* 2014; **103**: 137–49.

3 World Health Organization (WHO). Diabetes Key Facts. 2020. https://www.who.int/news-room/fact-sheets/detail/diabetes (accessed Sept 20, 2020).

4 Cho NH, Shaw JE, Karuranga S, *et al.* IDF Diabetes Atlas: Global estimates of diabetes prevalence for 2017 and projections for 2045. *Diabetes Res Clin Pract* 2018; **138**: 271–81.

5 Hussain A, Claussen B, Ramachandran A, Williams R. Prevention of type 2 diabetes: A review. *Diabetes Res Clin Pract* 2007; **76**: 317–26.

6 R.Jayawardena, P.Ranasinghe, N.M.Byrne, Soares MJ, Katulanda P, Hills AP. Prevalence and trends of the diabetes epidemic in South Asia: a systematic review and meta-analysis. *BMC Public Health* 2012; **12**: 380.

7 IDF Diabetes Atlas 9th edition 2019. Country Reports—Nepal. https://www.diabetesatlas.org/en/ (accessed Nov 19, 2019).

8 Gyawali B, Sharma R, Neupane D, Mishra SR, van Teijlingen E, Kallestrup P. Prevalence of type 2 diabetes in Nepal: A systematic review and meta-analysis from 2000 to 2014. *Glob Health Action* 2015; **8**. DOI:10.3402/gha.v8.29088.

9 Dhimal M, Karki KB, Sharma SK, *et al.* Prevalence of Selected Chronic Non-Communicable Diseases in Nepal. *J Nepal Health Res Counc* 2019; **17**: 394–401.

10 Bhandari GP, Angdembe MR, Dhimal M, Neupane S, Bhusal C. State of non-communicable diseases in Nepal. *BMC Public Health* 2014; **14**. DOI:10.1186/1471-2458-14-23.

11 Kafle N, Poudel R, Shrestha S. Noncompliance to Diet and Medication among Patients with Type 2 Diabetes Mellitus in Selected Hospitals of Kathmandu, Nepal. *J Soc Heal Diabetes* 2018; **06**: 090–5.

12 Lim H, Park J-E, Choi Y-J, Huh K-B, Kim W-Y. Individualized diabetes nutrition education improves compliance with diet prescription. *Nutr Res Pract* 2009; **3**: 315.

13 Forouhi NG, Misra A, Mohan V, Taylor R, Yancy W. Dietary and nutritional approaches for prevention and management of type 2 diabetes. *BMJ* 2018; **361**: 1–9.

14 Zheng F, Liu S, Liu Y, Deng L. Effects of an outpatient diabetes self-management education on patients with type 2 diabetes in China: A randomized controlled trial. *J Diabetes Res* 2019; **2019**. DOI:10.1155/2019/1073131.

15 Norris SL, Engelgau MM, Narayan KMV. Effectiveness of self-management training in type 2 diabetes: A systematic review of randomized controlled trials. *Diabetes Care* 2001; **24**: 561–87.

16 Mbhenyanea VC and XG. Dietary Management Practices for Diabetes by Dietitians in Public Hospitals in Limpopo Province , South Africa. *J Nutr Heal* 2017; **3**: 1–8.

17 Trento M, Basile M, Borgo E, *et al.* A randomised controlled clinical trial of nurse-, dietitian- and pedagogist-led Group Care for the management of Type 2 diabetes. *J Endocrinol Invest* 2008; **31**: 1038–42.

18 Huang MC, Hsu CC, Wang HS, Shin SJ. Prospective randomized controlled trial to evaluate effectiveness of registered dietitian-led diabetes management on glycemic and diet control in a primary care setting in Taiwan. *Diabetes Care* 2010; **33**: 233–9.

19 Schulz KF, Altman DG, Moher D. CONSORT 2010 Statement: Updated guidelines for reporting parallel group randomised trials. *BMJ* 2010; **340**: 698–702.

20 Muchiri JW, Gericke GJ, Rheeder P. Effect of a nutrition education programme on clinical status and dietary behaviours of adults with type 2 diabetes in a resource-limited setting in South Africa: A randomised controlled trial. *Public Health Nutr* 2016; **19**: 142–55.

21 Morris E, Aveyard P, Dyson P, *et al.* Dietary Approaches to the Management of type 2 Diabetes (DIAMOND): Protocol for a randomised feasibility trial. *BMJ Open* 2019; **9**: 1–9.

22 Ministry of Health, New ERA, The DHS Program ICF (2017). Nepal Demographic and Health Survey. 2016. https://www.dhsprogram.com/pubs/pdf/FR336/FR336.pdf (accessed July 11, 2019).

23 Nepal Health Research Council. Population Based Prevalence of Selected Non-Communicable Disease in Nepal. 2019 http://www.ghbook.ir/index.php?namecom_dbook&task=readonline&book_id=13650&page=73&chkhashk=ED9C9491B4&Itemid=218&lang=fa&tmpl=component.

24 Polonsky WH, Anderson BJ, Lohrer PA, *et al.* Assessment of diabetes-related distress. *Diabetes Care* 1995; **18**: 754–60.

25 JT F, MM F, GE H, *et al.* The reliability and validity of a brief diabetes knowledge test. *Diabetes Care* 1998; **21**: 706-710 5p.

26 Classification I. Standards of medical care in diabetes-2014. *Diabetes Care* 2014; **37**: 14–80.

# **Informed Consent to Participate in a Research Study**

| **Study Title:** | Effectiveness of Dietitian-Led Dietary Approach to Management of Diabetes in a Tertiary Care Hospital: A Randomized Control Trail |
| --- | --- |
| **Principal Investigator:** | Devendra Raj Singh  Contact number: 9851236658  Email: [dsingh3797@gmail.com](mailto:dsingh3797@gmail.com) |
| **Co-Investigator:** | Dev Ram Sunuwar  Contact number: 9851147339  Email: [devramsunuwar@gmail.com](mailto:devramsunuwar@gmail.com) |
| **Co-Investigator:** | Rajendra Lamichhane  Contact number: 9841736095  Email: [rajendralamichhane14@gmail.com](mailto:rajendralamichhane14@gmail.com) |
| **Funder:** | University Grant Commission (UGC) |

## **Statement**:

You are being invited to participate in a research study. This consent form will provide you with information on the research project, what you will need to do, and the associated risks and benefits of the research. Your participation is voluntary. Please read this form carefully. It is important that you ask questions and fully understand the research in order to make an informed decision. You will receive a copy of this document to take with you.

## **Purpose:**

The main purpose of this study is to examine the effectiveness of dietitian-led dietary approach to management of diabetes on reduction of HbA1c level, nutrition education score and macronutrient intake among T2DM patients in a tertiary care hospital in Kathmandu, Nepal.

## **Procedures:**

We are conducting a research study, and we intend to assess whether educating and prescribed diet plan will improve blood glucose levels and management of diabetes among diabetic patients. Your participation is voluntary. Your participants in this study will last about six months. You may decline to answer any question which you may not feel comfortable. You may end the interview at any time or refuse to participate in the study entirely. If you decide to participate in this study, you will be asked to respond to questionnaire on socio-demographic information, lifestyle related factors, dietary intake, and knowledge on diabetes. We will also take your 5 ml blood for biochemical test. We will also measure your height, weight and waist hip circumference. This will be taking one hour and will be repeated at the end of the study. Throughout the study, you will be called to follow up every 8 weeks in the hospital and also receive follow-up by phone calls to give your information and remind you about your eating habit.

## **Benefits:**

It is expected that you will learn about the role of diet, and how to eat well for you to control blood sugar levels. Also, this study shed light on the role of dietitians in the management of diabetes. These study findings are also expected to inform others who want to management of diabetes by nutrition education, counseling, and healthy diets.

## **Risks and Discomforts:**

Since this study is based on an educational trial, there is no harm to taking part in this study. Although you may feel a little discomfort while taking the blood sample. In addition to your time and inconvenience, there is the possibility that you may become uncomfortable answering the questions.

## **Payment/reimbursement:**

The research team will pay the lab test cost for all participants.

## **Privacy and Confidentiality:**

Your information will be strictly confidential and protected to the best of our ability. You will not be named in any report

## **Voluntary Participation:**

Taking part in this research study is entirely up to you. You may choose not to participate or you may discontinue your participation at any time without penalty or loss of benefits to which you are otherwise entitled. You will be informed of any new, relevant information that may affect your health, welfare, or willingness to continue your study participation.

## **Contact for additional information:**

If you do have any query regarding this study please feel free to ask. So, you are encouraged to ask any questions at any time of the study. For answers to any questions about the research, you may contact to Devendra Raj Singh (mobile number 9851236658; E-mail: [dsingh3797@gmail.com](mailto:dsingh3797@gmail.com)), and Dev Ram Sunuwar (mobile number 9851147339; E-mail: [devramsunuwar@gmail.com](mailto:devramsunuwar@gmail.com))

## **Consent Statement and Signature:**

I have read this consent form and have had the opportunity to have my questions answered to my satisfaction. I voluntarily agree to participate in this study. I understand that a copy of this consent will be provided to me for future reference.

________________________________ _____________________

**Participant Signature Date**

**Main Study Questionnaire in English - Baseline/End line**

| **Participants Information** | | | |
| --- | --- | --- | --- |
|  | Response | | Code |
| Study Group | Control 0  Intervention 1 | |  |
| Date |  | |  |
| Participants ID |  | | I1 |
| Participants full name |  | | I2 |
| Contact number |  | | I3 |
| Baseline interview date |  | | I4 |
| Follow-up date |  | | I5 |
| End line interview date |  | | I6 |
| Consent has been read and obtained | Yes  No | 1  2  If no, End | I7 |
| **Section 1: Socio-demographic information, Baseline** | | | |
| Sex ( record male/female as observed) | Male  Female | 1  2 | C1 |
| How old are you | …….years |  | C2 |
| What is your education level? | No formal education  Primary (1-5)  Secondary (6-10)  Higher secondary(11-12)  Bachelors and above  Refused | 1  2  3  4  5  88 | C3 |
| What is your ethnic background?  (Refer to caste classification: CC1 ) | Dalit  Disadvantaged Janajati  Relatively advantaged Janajati  Disadvantaged Non-dalit Terai caste group  Religious minorities  Upper caste group  Others  Refused | 1  2  3  4  5  6  7  88 | C4 |
| What is your religion? | Hindu  Buddhist  Muslim  Kirat  Christian  Others  Refused | 1  2  3  4  5  6  88 | C5 |
| What is your marital status? | Never married  Married  Separated/divorced  Widowed  Cohabiting  Refused | 1  2  3  4  5  88 | C6 |
| Which of the following best describes your main occupation? | Government employee  Non-government employee  Self-employed  Students  Homemaker  Retired  Unemployed  Others  Refused | 1  2  3  4  5  6  7  8  88 | C7 |
| Where do you live? | Urban  Rural | 1  2 | C8 |
| How much is your average monthly Income? | Rs | ……… | C9 |
| Family history of Diabetes Mellitus | Yes  No | 1  2 | C10 |
| **Section 2: Behavioral information Baseline/Endline** | | | |
| **Tobacco use** | | | |
| Do you currently smoke any tobacco products, such as cigarettes, bidis, cigars, pipes, hukahs, or tamakhus? | Yes  No | 1  2  If No, go to A1 | T1 |
| How frequently do you smoke tobacco products? | Daily  5-6 days a week  3-4 days a week  1-2 days a week  Occasionally | 1  2  3  4  5 | T2 |
| In the past, did you ever smoke any tobacco products? | Yes  No | 1  2  If No, go to A1 | T3 |
| In the past, how frequently did you smoke tobacco products? | Daily  5-6 days a week  3-4 days a week  1-2 days a week  Occasionally | 1  2  3  4  5 | T4 |
| **Alcohol consumption** | | | |
| Have you **ever** consumed an alcoholic drink such as beer, wine, spirits fermented cider or *jaad, chyang, raksi, aila or tungba?* | Yes  No | 1  2  If No, go to P1a | A1 |
| Have you consumed an alcoholic drink within the past 12 months? | Yes  No | 1  2 | A2 |
| During the past 12 months, how frequently have you had at least one standard alcoholic drink? | Daily  5-6 days per week  3-4 days per week  1-2 days per week  1-3 days per month  Less than once a month | 1  2  3  4  5  6 | A3 |
| **Section 3: Physical and biochemical measurement** | | | |
| Blood pressure |  |  |  |
| Reading 1 | Systolic (mmHg) | ……. | P1a |
|  | Diastolic (mmHg) | ……. | P1b |
|  | Beats per minute | ……. | P2a |
| Reading 2 | Systolic (mmHg) | ……. | P4a |
|  | Diastolic (mmHg) | ……. | P2b |
|  | Beats per minute | ……. | P4b |
| Reading 3 | Systolic (mmHg) | ……. | P3a |
|  | Diastolic (mmHg) | ……. | P4b |
|  | Beats per minute | …… | P4c |
| **Height, weight, waist and Hip Circumference** | | | |
| Height | in centimeter (cm) | …….. | P5 |
| Weight | in kilograms (kg) | …….. | P6 |
| Waist Circumference | in centimeter (cm) | …….. | P7 |
| Hip circumferences | in centimeter (cm) | …….. | P8 |
| **Biochemical measurements** | | | |
| HbA1c | % | ........ | P9 |
| FBS | mg/dL | …… | P10 |
| Total cholesterol | mg/dL | …… | P11 |
| Triglycerides | mg/dL | …… | P12 |
| LDL | mg/dL | …… | P13 |
| HDL | mg/dL | …… | P14 |
|  |  |  |  |
| **Current medication used** | | | |
|  |  |  | M1 |
|  |  |  | M2 |
|  |  |  | M3 |
|  |  |  | M4 |
|  |  |  | M5 |
|  |  |  | M6 |
|  |  |  | M7 |
|  |  |  | M8 |
|  |  |  | M9 |
|  |  |  | M10 |
|  |  |  | M11 |
|  |  |  | M12 |
|  |  |  | M13 |
|  |  |  | M14 |

**Section 4: A 24 hour recall, Baseline/End line**

|  | **Items** | **Description of food or drinks** | **Household amount** | **Amount (g or ml)** | **Preparation/ingredients** |
| --- | --- | --- | --- | --- | --- |
| **Breakfast** |  |  |  |  |  |
|  |  |  |  |  |  |
|  |  |  |  |  |  |
|  |  |  |  |  |  |
|  |  |  |  |  |  |
|  |  |  |  |  |  |
|  |  |  |  |  |  |
| **Lunch** |  |  |  |  |  |
|  |  |  |  |  |  |
|  |  |  |  |  |  |
|  |  |  |  |  |  |
|  |  |  |  |  |  |
|  |  |  |  |  |  |
|  |  |  |  |  |  |
|  |  |  |  |  |  |
|  |  |  |  |  |  |
|  |  |  |  |  |  |
| **Snacks/mid-day** |  |  |  |  |  |
|  |  |  |  |  |  |
|  |  |  |  |  |  |
|  |  |  |  |  |  |
|  |  |  |  |  |  |
|  |  |  |  |  |  |
|  |  |  |  |  |  |
|  |  |  |  |  |  |
|  |  |  |  |  |  |
|  |  |  |  |  |  |
|  |  |  |  |  |  |
| **Dinner** |  |  |  |  |  |
|  |  |  |  |  |  |
|  |  |  |  |  |  |
|  |  |  |  |  |  |
|  |  |  |  |  |  |
|  |  |  |  |  |  |
|  |  |  |  |  |  |

**Section 5 Quality of life: The Problem Areas in Diabetes (PAID) score, Baseline/End line**

| **SN** | **Questions** | **Not a problem** | **Minor problem** | **Moderate problem** | **Somewhat serious problem** | **Serious problem** |
| --- | --- | --- | --- | --- | --- | --- |
| 1 | Not having clear and concrete goals for your diabetes care? | 0 | 1 | 2 | 3 | 4 |
| 2 | Feeling discouraged with your diabetes treatment plan? | 0 | 1 | 2 | 3 | 4 |
| 3 | Feeling scared when you think about living with diabetes? | 0 | 1 | 2 | 3 | 4 |
| 4 | Uncomfortable social situations related to your diabetes care (e.g. people telling you what to eat)? | 0 | 1 | 2 | 3 | 4 |
| 5 | Feeling of deprivation regarding food and meals? | 0 | 1 | 2 | 3 | 4 |
| 6 | Feeling depressed when you think about living with diabetes? | 0 | 1 | 2 | 3 | 4 |
| 7 | Not knowing if your mood or feelings are related to your diabetes? | 0 | 1 | 2 | 3 | 4 |
| 8 | Feeling overwhelmed by your diabetes? | 0 | 1 | 2 | 3 | 4 |
| 9 | Worrying about low blood sugar reactions? | 0 | 1 | 2 | 3 | 4 |
| 10 | Feeling angry when you think about living with diabetes? | 0 | 1 | 2 | 3 | 4 |
| 11 | Feeling constantly concerned about food and eating? | 0 | 1 | 2 | 3 | 4 |
| 12 | Worrying about the future and the possibility of serious complication? | 0 | 1 | 2 | 3 | 4 |
| 13 | Feeling of guilt or anxiety when you get off tract with your diabetes management? | 0 | 1 | 2 | 3 | 4 |
| 14 | Not "accepting" your diabetes? | 0 | 1 | 2 | 3 | 4 |
| 15 | Feeling unsatisfied with your diabetes physician? | 0 | 1 | 2 | 3 | 4 |
| 16 | Feeling the diabetes is taking up too much of your mental and physical energy every day? | 0 | 1 | 2 | 3 | 4 |
| 17 | Feeling alone with your diabetes? | 0 | 1 | 2 | 3 | 4 |
| 18 | Feeling that your friends and family are not supportive of your diabetes management efforts? | 0 | 1 | 2 | 3 | 4 |
| 19 | Coping with complications of diabetes? | 0 | 1 | 2 | 3 | 4 |
| 20 | Feeling "burned out" by the constant effort needed to manage diabetes? | 0 | 1 | 2 | 3 | 4 |
|  |  |  |  |  |  |  |

**Section 6: Diabetes Knowledge Questionnaire (DKQ), Baseline/End line**

| **Items** | **Questions** | **Yes** | **No** | **I don't know** |
| --- | --- | --- | --- | --- |
| 1 | Eating too much sugar and other sweet foods is a cause of diabetes. |  |  |  |
| 2 | The usual cause of diabetes is lack of effective insulin in the body. |  |  |  |
| 3 | Diabetes is caused by failure of the kidneys to keep sugar out of the urine. |  |  |  |
| 4 | Kidneys produce insulin |  |  |  |
| 5 | In untreated diabetes, the amount of sugar in the blood usually increase. |  |  |  |
| 6 | If I am debates, my children have a higher chance of being diabetic |  |  |  |
| 7 | Diabetes can be cured. |  |  |  |
| 8 | A fasting blood sugar level of 210 is too high. |  |  |  |
| 9 | The best way to check my diabetes is by testing my urine. |  |  |  |
| 10 | Regular exercise will increase the need for insulin or other diabetes medication. |  |  |  |
| 11 | There are two main types of diabetes: Type 1 (insulin-dependent) and Type 2 (non-insulin-dependent) |  |  |  |
| 12 | An insulin reaction is caused by too much food. |  |  |  |
| 13 | Medication is more important than diet and exercise to control my diabetes. |  |  |  |
| 14 | Diabetes often causes poor circulation |  |  |  |
| 15 | Cuts and abrasions on diabetic heal more slowly. |  |  |  |
| 16 | Diabetics should take extra care when cutting their toenails. |  |  |  |
| 17 | A person with diabetes should cleanse a cut with iodine and alcohol |  |  |  |
| 18 | The way I prepare my food is as important as the foods I eat. |  |  |  |
| 19 | Diabetes can damage my kidneys |  |  |  |
| 20 | Diabetes can cause loss of feeling in my hands, fingers, and feet |  |  |  |
| 21 | Shaking and sweating are signs of high blood sugar |  |  |  |
| 22 | Frequent urination and thirst are signs of low blood sugar. |  |  |  |
| 23 | Tight elastic hose or socks are not bad for diabetics. |  |  |  |
| 24 | A diabetic diet consists mostly of special foods |  |  |  |

**Section 7: Perceived Dietary Adherence Questionnaire (PDAQ), Baseline/End line**

| **SN** | **Questions** | **0** | **1** | **2** | **3** | **4** | **5** | **6** | **7** |
| --- | --- | --- | --- | --- | --- | --- | --- | --- | --- |
| 1 | On how many of the last SEVEN DAYS have you followed a healthful eating plan? |  |  |  |  |  |  |  |  |
| 2 | On how many of the last SEVEN DAYS did you eat the number of fruit and vegetables? |  |  |  |  |  |  |  |  |
| 3 | On how many of the last SEVEN DAYS did you eat carbohydrate-containing foods with a low Glycemic Index? (examples: dried beans, lentils, barley, pasta, low fat dairy products) |  |  |  |  |  |  |  |  |
| 4 | On how many of the last SEVEN DAYS did you eat foods high in sugar, such as cakes, cookies, desserts, candies? |  |  |  |  |  |  |  |  |
| 5 | On how many of the last SEVEN DAYS did you eat foods high in fibre such as oatmeal, high fibre cereals, and whole-grains breads? |  |  |  |  |  |  |  |  |
| 6 | On how many of the last SEVEN DAYS did you space carbohydrate evenly throughout the day? |  |  |  |  |  |  |  |  |
| 7 | On how many of the last SEVEN DAYS did you eat fish or other foods high in omega-3 fats? |  |  |  |  |  |  |  |  |
| 8 | On how many of the last SEVEN DAYS did you eat foods that contained or was prepared with cannola, walnut, olive, or flax oils? |  |  |  |  |  |  |  |  |
| 9 | On how many of the last SEVEN DAYS did you eat foods high in fat (such as high fat dairy products, fatty meat, fried foods or deep fried foods)? |  |  |  |  |  |  |  |  |

| **Section 8: Physical activity** | | | | |
| --- | --- | --- | --- | --- |
| I am going to ask you about the time you spend doing different types of physical activity in a typical week. Please answer these questions even if you do not consider yourself to be a physically active person.  In answering the following questions 'vigorous-intensity activities' are activities that require hard physical effort and cause large increases in breathing or heart rate, 'moderate-intensity activities' are activities that require moderate physical effort and cause small increases in breathing or heart rate. | | | | |
| **Work** | **Response** | | **code** | **Skip/Remarks** |
| Does your work involve vigorous-intensity activity that causes large increases in breathing or heart rate like [carrying or lifting heavy loads, digging or construction work] for at least 10 minutes continuously?  (USE SHOWCARDS 1a) | Yes  No | 1  2 | P1 | If No, go to p4 |
| In a typical week, on how many days do you do vigorous intensity activities as part of your work? | Number of day | ……….. | P2 |  |
| How much time do you spend doing vigorous-intensity activities at work on a typical day? | Hours  Min | ……….  ............. | P3  (a-b) |  |
| Does your work involve moderate-intensity activity that causes small increases in breathing or heart rate such as brisk walking [or carrying light loads] for at least 10 minutes continuously? (USE CHOWCARDS 1b) | Yes  No | 1  2 | P4 | If No, go to P 7 |
| In a typical week, on how many days do you do moderate intensity activities as part of your work? | Number of day | ............... | P5 |  |
| How much time do you spend doing moderate-intensity activities at work on a typical day? | Hours  Min | ...............  ............... | P6  (a-b) |  |
| **Travel to and from places** | | | | |
| Now, I would like to ask you about the usual way you travel to and from places. For example to work, for shopping, to market, to place of | | | | |
| **Work** | **Response** | | **code** | **Skip/Remarks** |
| Do you walk or use a bicycle (pedal cycle) for at least 10 minutes continuously to get to and from places? | Yes  No | 1  2 | P7 | If No, go to P 10 |
| In a typical week, on how many days do you walk or bicycle for at least 10 minutes continuously to get to and from places? | Number of day | ............... | P8 |  |
| How much time do you spend walking or bicycling for travel on a typical day? | Hours  Min | ...............  ............... | P9  (a-b) |  |
| **Recreational activities** | | | | |
| Now I would like to ask you about sports, fitness and recreational activities | | | | |
| **Work** | **Response** | | **code** | **Skip/Remarks** |
| Do you do any vigorous-intensity sports, fitness or recreational (leisure) activities that cause large increases in breathing or heart rate like [running or football] for at least 10 minutes continuously? (USE SHOWCARDS 1c) | Yes  N) | **1**  2 | P10 | If No, go to P 13 |
| In a typical week, on how many days do you do vigorous intensity sports, fitness or recreational (leisure) activities? | Number of day | ............... | P11 |  |
| How much time do you spend doing vigorous-intensity sports, fitness or recreational activities on a typical day? | Hour  Min | ...............  ............... | P12  (a-b) |  |
| Do you do any moderate-intensity sports, fitness or recreational (leisure) activities that cause a small increase in breathing or heart rate such as brisk walking, [cycling, swimming, and volleyball] for at least 10 minutes continuously?  (USE SHOWCARDS 1d) | Yes  No | 1  2 | P13 | If No, go to P16 |
| In a typical week, on how many days do you do moderate intensity sports, fitness or recreational (leisure) activities? | Number of day | ............... | P14 |  |
| How much time do you spend doing moderate-intensity sports, fitness or recreational (leisure) activities on a typical day? | Hours  Min | ...............  ............... | P15  (a-b) |  |
| **Sedentary behavior** | | | | |
| The following question is about sitting or reclining at work, at home, getting to and from places, or with friends including time spent sitting at a desk, sitting with friends, traveling in car, bus, train, reading, playing cards or watching television, but do not include time spent sleeping (USE SHOWCARDS 1e) | | | | |
| How much time do you usually spend sitting or reclining on a typical day? | Hours  Min | ...............  ............... | P16  (a-b) |  |

**DIAM-D show cards**

**CC1: Cast classification card**

| **Dalit** | **Dalit** | **Disadvantaged**  **Janajati** | **Disadvantaged**  **Janajati** | **Disadvantaged**  **Non-Dalit Terai caste groups** | **Religious minorities** | **Relatively advantaged Janajaties** | **Upper caste groups** |
| --- | --- | --- | --- | --- | --- | --- | --- |
| **Hill** | **Terai** | **Hill** | **Terai** |  |  |  |  |
| Badi | Bantar | Baramu | Dhanged/Jhanged | Badhe | Churoute | Gurung | Baniya |
| Damai | Chamar | Bhote | Dhanuk | Bhediyar | Muslims | Thakali | Bengali |
| Gaine | Chaidimar | Bote | Dhimal | Bing/Banda |  | Newar | Brahman (hill) |
| Sarki | Dhobi | Byansi | Gangal | Dhunia |  |  | Brahman (Terai) |
| Kami | Dom | Chepang | Kisan | Hajam/Thakur |  |  | Chhetri |
|  | Dusah | Chhantal | Koche | Haluwati |  |  | Jainekayastha |
|  | Halkhor | Danuwar | Meche | Kahar |  |  | Marwadi |
|  | Khatway | Derai | Munda | Kalwar |  |  | Nuraang |
|  | Mushar | Dura | Pattarkatta/Kusbadiay | Kanu |  |  | Rajput |
|  | Paswan | Gari/Bhujel | Rajbanshi | Kewat |  |  | Sanyasi |
|  | Tatma | Hayu | Santhal/Satar | Koiri |  |  | Thakuri |
|  |  | Hyolomo | Tajpuria | Kumar |  |  |  |
|  |  | Jirel | Tharu | Kumhar |  |  |  |
|  |  | Kusunda |  | Kurmi |  |  |  |
|  |  | Kumal |  | Lodhar |  |  |  |
|  |  | Lepcha |  | Mali |  |  |  |
|  |  | Limbu |  | Mallah |  |  |  |
|  |  | Magar |  | Nuniya |  |  |  |
|  |  | Majhi |  | Rajba |  |  |  |
|  |  | Pahari |  | Sonar |  |  |  |
|  |  | Rai |  | Sudhi |  |  |  |
|  |  | Raji |  | Teli |  |  |  |
|  |  | Raute |  | Yadav |  |  |  |
|  |  | Sherpa |  |  |  |  |  |
|  |  | Sunuwar |  |  |  |  |  |
|  |  | Tamang |  |  |  |  |  |
|  |  | Thami |  |  |  |  |  |
|  |  | Walung |  |  |  |  |  |
|  |  | Yakkah |  |  |  |  |  |

**Physical activity**

| **1a: Vigorous physical activity at work** | **1b: Moderate physical activity at work** | **1c: Vigorous physical activity during leisure time** | **11d: Moderate physical activity during leisure time** | **11e: Sedentary behavior** |
| --- | --- | --- | --- | --- |
| Make you breathe much harder than normal   - Ploughing field and digging ditch - Constructive work - Very hard labor work - Carrying or lifting heavy loads - Cycle rickshaw driving | Make you breathe somewhat harder than normal   - Washing clothes - Gardening - Lifting light weights - Mopping floor - Brisk walking | Make you breathe much harder than normal   - Most competitive sports: Playing football, Basketball, Rugby - Running - Skipping - Martial arts | - Volleyball - Cricket - Cycling - Swimming - Yoga - Badminton - Brisk walking - Weight training and body building using free weights | - Sitting at desk - Sitting with friends - Traveling in car or bus - Reading - Playing cards - Watching television |
